# Non-invasive characterization of human bone marrow stimulation and reconstitution by cell-free messenger RNA sequencing

**DOI:** 10.1038/s41467-019-14253-4

**Published:** 2020-01-21

**Authors:** Arkaitz Ibarra, Jiali Zhuang, Yue Zhao, Neeraj S. Salathia, Vera Huang, Alexander D. Acosta, Jonathan Aballi, Shusuke Toden, Amy P. Karns, Intan Purnajo, Julianna R. Parks, Lucy Guo, James Mason, Darren Sigal, Tina S. Nova, Stephen R. Quake, Michael Nerenberg

**Affiliations:** 1 Molecular Stethoscope, Inc., 3210 Merryfield Row, San Diego, CA 92121 USA; 2 Scripps Clinic Medical Group, Scripps Green Hospital, 10666 N Torrey Pines Road, La Jolla, CA 92037 USA; 3 Department of Bioengineering and Department of Applied Physics, Stanford University and Chan Zuckerberg Biohub, 318 Campus Drive, Stanford, CA 94305 USA

**Keywords:** Gene expression analysis, Sequencing, RNA sequencing, Molecular medicine

## Abstract

Circulating cell-free mRNA (cf-mRNA) holds great promise as a non-invasive diagnostic biomarker. However, cf-mRNA composition and its potential clinical applications remain largely unexplored. Here we show, using Next Generation Sequencing-based profiling, that cf-mRNA is enriched in transcripts derived from the bone marrow compared to circulating cells. Further, longitudinal studies involving bone marrow ablation followed by hematopoietic stem cell transplantation in multiple myeloma and acute myeloid leukemia patients indicate that cf-mRNA levels reflect the transcriptional activity of bone marrow-resident hematopoietic lineages during bone marrow reconstitution. Mechanistically, stimulation of specific bone marrow cell populations in vivo using growth factor pharmacotherapy show that cf-mRNA reflects dynamic functional changes over time associated with cellular activity. Our results shed light on the biology of the circulating transcriptome and highlight the potential utility of cf-mRNA to non-invasively monitor bone marrow involved pathologies.

## Abstract (web summary)

Circulating cell-free mRNA holds great promise as a non-invasive diagnostic biomarker. Here the authors show that cell-free mRNA captures transcripts from the bone marrow and can be used to non-invasively monitor dynamic changes in bone marrow physiology.

## Introduction

Blood irrigates all organs, supplying oxygen and nutrients to the cells of the body while collecting byproducts of cell metabolism, including lipids, proteins and nucleic acids. These circulating biomolecules in blood contain information linked to specific organ health. While most research effort has focused on circulating proteins and lipids, circulating cell-free nucleic acids (cf-NA) have recently emerged as a non-invasive tool for diagnosis and monitoring of health and disease^1^. For example, cell-free DNA (cfDNA), the most well-characterized cf-NA, has been utilized for prenatal diagnostics, transplant rejection prediction, and monitoring of cancer^2–6^. Despite these advances, the value of cfDNA tests remains mainly restricted to physiologic and disease situations characterized by genetic differences (i.e., pregnancy, transplants, or tumors). In contrast, the cell-free messenger RNA (cf-mRNA) transcriptome can be considered as a compendium of transcripts collected from all organs^7^. Some of these circulating transcripts correspond to well-characterized tissue-specific genes, supporting interrogation of these biomolecules to dynamically monitor health or disease state of tissues and organs. Indeed, cf-mRNA has been shown to reflect fetal development, predict preterm delivery in pregnant women^7–9^, and as a cancer biomarker^10,11^.

Strikingly, the biological processes underlying the presence of cf-NA in circulation remain inferred, but largely unknown. In the case of cfDNA, studies have proposed the primary mechanism is passive release into circulation upon cell death^12,13^. In contrast, RNA molecules can be actively secreted from cells^11,14–16^. Much work has focused on the secretion of non-coding and smaller RNA molecules into exosomes and other lipid vesicles. However, on a per-molecule basis, mRNA comprises a minor fraction of this phenomenon^17^, and the origin of cf-mRNA remains unclear.

In this study, we conduct next-generation sequencing-based whole-transcriptomic profiling of cf-mRNA and compare expression levels to those from circulating cells of the blood (CC) to decipher the origin of circulating transcripts and better understand their potential clinical utility. We show that cf-mRNA captures transcripts of non-hematopoietic and hematopoietic origin, and is enriched in transcripts derived from the bone marrow (BM). Longitudinal studies of cancer patients undergoing BM ablation and transplantation show that cf-mRNA profiling non-invasively captures temporal transcriptional activity of the BM. Further, stimulation of specific BM lineages with growth factor therapeutics suggests that cf-mRNA fluctuations reflect active lineage-specific transcriptional activity. Collectively, our data provide insights into the biological origins of cf-mRNA, strongly suggesting that living cells contribute cf-mRNA to circulation, and anticipate the potential of circulating transcripts as non-invasive biomarkers that could eventually alleviate the use of BM biopsies.

## Results

### cf-mRNA is enriched in hematopoietic progenitor transcripts

**Fig. 1:**
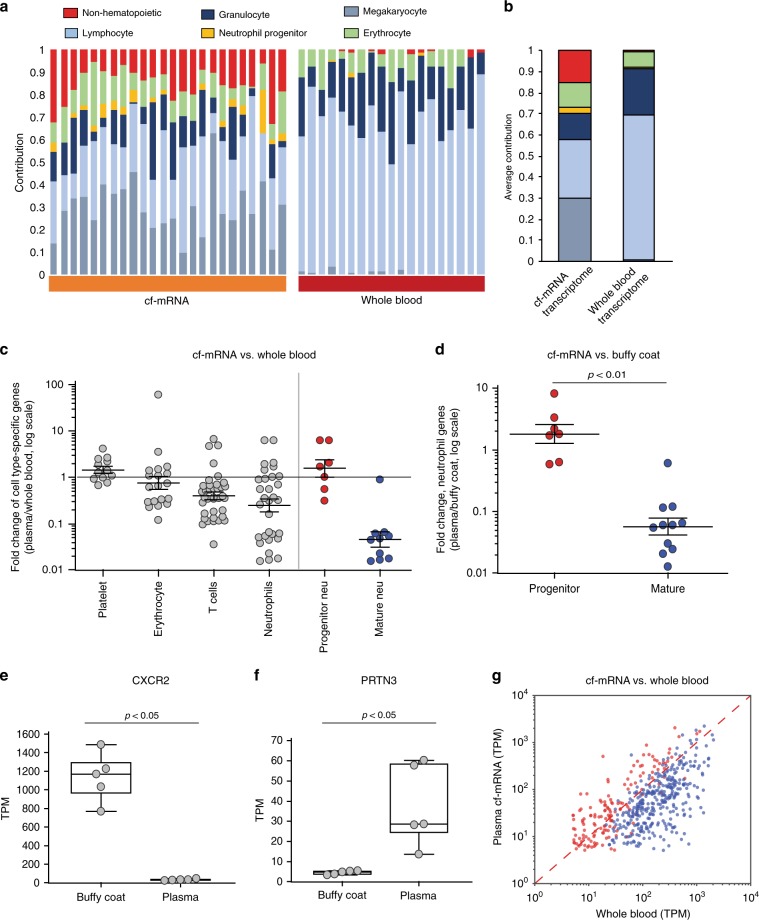
cf-mRNA transcriptome captures hematopoietic transcripts derived from the bone marrow. **a** cf-mRNA transcriptome and whole-blood transcriptome from healthy subjects was decomposed using non-negative matrix factorization and tissue contribution estimated using public databases. cf-mRNA was sequenced from 24 normal donors and whole-blood RNA-Seq data from 19 healthy individuals reported in Nguyen et al. ^20^. Estimated contribution of the indicated cell types/tissues for each sample is shown. **b** Average values for each biofluid (24 cf-mRNA and 19 whole-blood samples) are shown using the same color code. **c**. RNA-Seq was performed in three paired plasma and whole-blood samples from healthy individuals. Levels of detectable cell-type-specific transcripts (Supplementary Table 5) were compared between cf-mRNA and whole blood for all three donors. Average fold change (cf-mRNA/whole blood) among the three individuals is represented (log scale) (two-sided Wilcoxon’s rank-sum test, *U* = 3.22). Red dots, neutrophil progenitor transcripts. Blue dots, mature neutrophil transcripts. Cell-type-specific genes were identified as explained in Methods. **d** RNA-Seq was performed in five paired plasma and buffy coat samples from healthy control individuals. Levels of mature and progenitor neutrophil transcripts detectable in plasma and matching buffy coat specimens were compared. Average fold change of these transcripts (plasma/buffy coat) in the five paired samples is shown (log scale). Two-sided Wilcoxon’s rank-sum test was performed (*U* = −3.40, *p* value = 0.00068). **e**, **f** Box-plot comparing the normalized levels (TPM) of the indicated transcripts in paired buffy coat and cf-mRNA samples measured by RNA-Seq (*n* = 5 samples, two-sided Wilcoxon’s rank-sum test), **f** PRTN3, *U* = −2.61, *p* value = 0.0090; **e** CXCR2, *U* = 2.61, *p* value = 0.0090. Center line, median; box limits, upper and lower quartiles; whiskers, 1.5× interquartile range; points, outliers. Source data for **b**–**f** are provided as a Source Data file **g**. Scatter plot comparing the levels in matching cf-mRNA (*Y* axis) and whole blood (*X* axis) of BM-specific genes (red dots) and peripheral blood-specific genes (blue dots), which form two distinct populations (*p* < 0.001, *U* = 18.58, two-sided Wilcoxon’s rank-sum test).

To characterize the landscape of the human cell-free RNA transcriptome (cf-mRNA), we isolated and sequenced cf-mRNA from 1 ml of serum of 24 healthy donors. Among this cohort, we identified 10,357 transcripts with >1 TPM (transcripts per million) and 7386 transcripts with >5 TPM in at least 80% of the samples, reflecting the diversity and consistency of cf-mRNA transcriptome among healthy subjects (Supplementary Tables 1 and 2 provide additional information of cf-mRNA sequencing metrics). We used non-negative matrix factorization (NMF) to decompose the cf-mRNA transcriptome in an unsupervised manner^18,19^ and gene expression reference databases (GTEx and Blueprint) to estimate the relative contributions of the different tissues and cell types (see Methods). The majority of the transcripts detected in cf-mRNA, ~85% on average, are of hematopoietic origin (i.e., derived from circulating cells and BM-resident cells), with the remaining ~15% being of non-hematopoietic origin (i.e., derived from solid tissues, Fig. 1a, b). Specifically, deconvolution analyses estimated that, on average, ~29% of transcripts are of megakaryocyte/platelet origin (first to third quartile range 23–36%), ~28% are of lymphocyte origin (range 18–30%), 12.8% of granulocyte origin (range 6–16%), 3% of neutrophil progenitor origin (range 0.2–3.7%), 11% of erythrocyte origin (range 8–14%), and ~15% derived from solid tissues (range 11–20%) (Fig. 1a, b). To gain insight into the origin of these transcripts, similar deconvolution analysis was performed in whole-blood (WB) samples from 19 healthy individuals from previously reported RNA-sequencing (RNA-Seq) data^20^. As expected, the WB transcriptome is largely composed of lymphocyte (~69% on average) and granulocyte (~22% on average) transcripts, with an additional ~7% of transcripts of erythrocyte origin and minor contributions from other cell types and tissues (Fig. 1a, b). These analyses represent an estimation of the composition of the transcriptome of these biofluids that could be influenced by different factors. Nevertheless, our data show the higher diversity of cf-mRNA transcriptome, which, compared to WB, contains a larger fraction of non-hematopoietic transcripts and of hematopoietic progenitor genes derived from the BM.

To confirm the presence of BM-specific transcripts in circulation, we performed RNA-Seq in three paired WB (which includes all cellular components of blood) and plasma samples from healthy donors (Supplementary Fig. 1A) and compared the levels of the main hematopoietic cell-type-specific transcripts (i.e., neutrophils, erythrocytes, platelets/megakaryocyte, T cells) in these specimens (Fig. 1c and Supplementary Fig. 1B, C). Striking differences were observed among neutrophil-specific transcripts (Fig. 1c). Using the hematopoiesis transcriptomic reference database (Blueprint), we observed that transcripts expressed in mature circulating neutrophils are detected at much lower levels in plasma compared to WB (Fig. 1c). In contrast, transcripts expressed in BM-resident neutrophil progenitors are enriched in cf-mRNA (Fig. 1c). To confirm these findings, we performed RNA-Seq of five paired plasma and buffy coat samples (buffy coat is enriched in white blood cells). Consistently, neutrophil mature and progenitor transcripts were found to form distinct populations (Fig. 1d), in which cf-mRNA shows low levels of mature transcripts such as the chemokine receptors CXCR1 and CXCR2 (Fig. 1e, *p* < 0.01) compared to buffy coat, but is enriched in progenitor transcripts, such as PRTN3 (myeloblastin precursor), CTSG (cathepsin G), and AZU1 (azurocidin precursor) (*p* < 0.05, Fig. 1f, Supplementary Fig. 1D, E). These data support the presence of BM transcripts in cf-mRNA; indeed, quadratic programing deconvolution analysis of hematopoietic transcripts from healthy donors indicated that BM transcripts contribute ~9% of cf-mRNA transcriptome, in contrast to ~1% in WB.

To further confirm this result, we performed RNA-Seq on a human BM sample and compared it with the WB transcriptome. We identified 377 genes enriched in BM transcriptome (>5-fold, BM genes) (Supplementary Table 3), representing hematopoietic progenitors (i.e., neutrophil progenitors and mesenchymal stem cells from the BM). Interestingly, progenitor transcripts such as PRTN3, CTSG, and AZU1 are among the top transcripts enriched in BM transcriptome. In addition, 374 genes were identified enriched in WB (>5-fold, WB genes) (Supplementary Table 4), mainly representing mature circulating blood cell genes (i.e., associated with mature granulocytes and lymphocytes). Subsequently, the levels of BM genes and WB genes were compared in three matching WB and plasma samples, which confirmed that these transcripts segregate into two populations (*p* < 0.001), with cf-mRNA being enriched in hematopoietic progenitor genes (BM genes) and depleted of mature genes (WB genes) compared to WB (Fig. 1g and Supplementary Fig. 1F). In summary, our data indicate that cf-mRNA transcriptome captures transcripts derived from the BM, potentially providing a window to non-invasively evaluate BM function

### Measurement of BM-specific transcripts by cf-mRNA

**Fig. 2:**
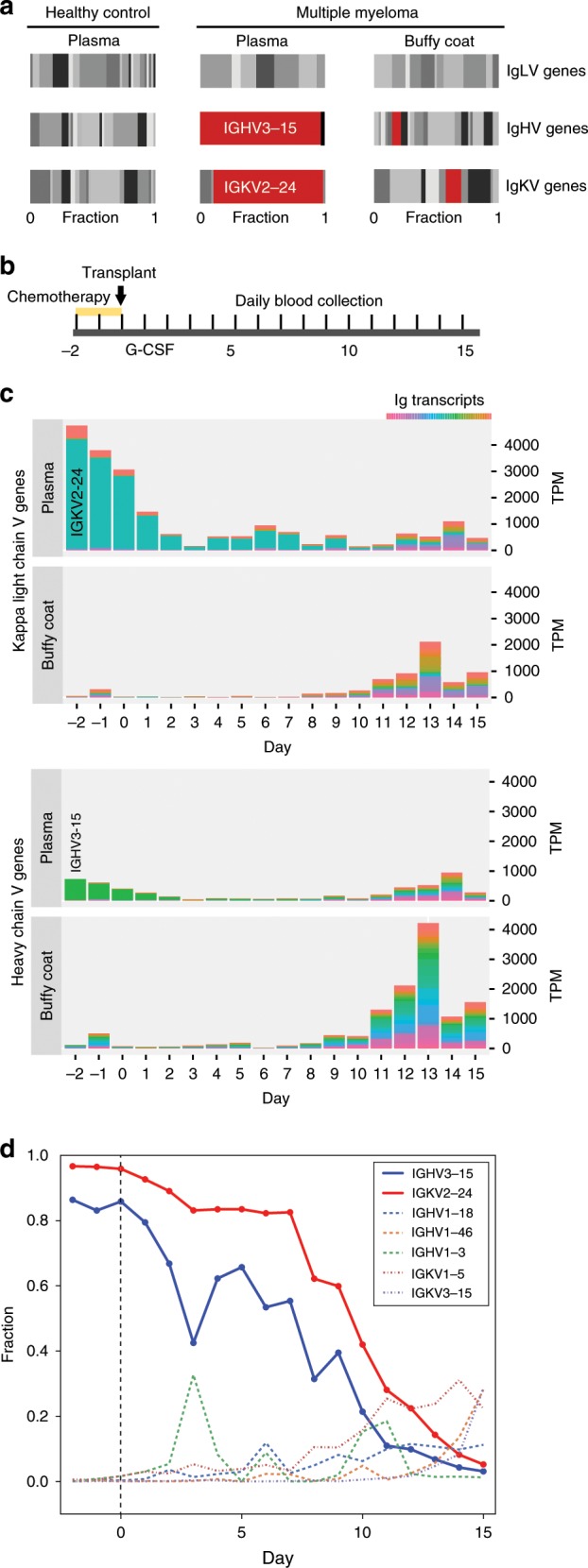
cf-mRNA transcriptome reflects dynamical changes of Ig transcripts derived from the BM. **a** Matching cf-mRNA and buffy coat samples from a multiple myeloma patient (day −2) were analyzed by RNA-Seq. Fraction of transcripts from the variable regions of the immunoglobulin heavy and light chains identified in plasma and buffy coat samples are shown (center and right panels). Clonally amplified transcripts are indicated in red and dominated the representation of Ig transcripts in cf-mRNA of the MM patient. Levels of Ig transcripts in plasma of a healthy individual (left panel) are shown as reference. **b** Schematic of the therapeutic treatment performed in MM patients. Melphalan-mediated BM ablation started at day −2, autologous stem cell transplant was performed at day 0. Steroids and G-CSF were then administered as supportive care. Blood was collected every day during the study. **c**. Bar graphs showing the normalized values (TPM, *Y* axis) of Ig transcripts detected by RNA-Seq in paired plasma and buffy coat samples throughout the treatment. The repertoire of variable regions of Ig heavy chain and Ig kappa light chain are shown in a color gradient. Dominant transcripts identified in plasma are indicated. Day of blood collection with respect to transplant is indicated in the *X* axis. **d** Fraction of transcripts from variable Ig regions in cf-mRNA during BM ablation and transplant. Day of blood collection with respect to transplant is indicated in the *X* axis. Dominant Ig transcripts, shown in solid blue and red lines, decrease after melphalan-mediated BM ablation.

As further evidence that BM-specific transcripts can be detected in cf-mRNA and to evaluate their potential utility, we recruited three multiple myeloma (MM) patients. MM is characterized by the clonal expansion and accumulation of malignant plasma cells almost exclusively in the BM. These cells express specific immunoglobulin (Ig) rearrangements, in contrast to plasma cells of healthy individuals, which express multiple Ig combinations. In this study, MM patients underwent melphalan-mediated BM ablation (starting at day −2), followed by autologous hematopoietic stem cell (HSC) infusion (day 0) (Fig. 2b). We isolated and sequenced cf-mRNA from 1 ml of plasma of these patients before BM ablation (day −2). Clonal expansion of Ig heavy (IgH) and Ig light (IgL) chains transcripts was identified for two out of three patients. For instance, in patient 2 we detected IGHG1 and IGKC transcripts as the most prevalent Ig constant regions (Supplementary Fig. 2A–C). For the variable regions, IGHV3–15 and IGKV2–24 transcripts dominated the sample’s transcriptome, while clonal lambda regions were not detected (Fig. 2a, c and Supplementary Fig. 2C). In contrast, clonal transcripts were not observed in plasma of a healthy control individual, as expected (Fig. 2a). Similar analyses in patient 1 revealed a clone composed of the IgH constant chain IGHA1 and variable region IGHV1–69, and IgL lambda constant chain IGLC1 and variable region IGLV1–40 (Supplementary Fig. 2D). In both cases, the malignant clones we identified are consistent with the molecular testing performed from BM aspirates (Supplementary Table 6). However, for patient 3, we did not detect dominant Ig rearrangements (Supplementary Fig. 2E), likely due to the low number of plasma cells in the BM of this patient at the start of this study (Supplementary Table 6). Malignant plasma cells are rarely found in circulation in MM patients; indeed, RNA-Seq analysis of the matching buffy coat of patient 2 samples before chemotherapy treatment showed only low levels of a repertoire of IgH and IgL transcripts, with no dominant rearrangements (Fig. 2a, c and Supplementary Fig. 2A–C), highlighting the unique ability of cf-mRNA to capture the clonal Ig transcripts generated by plasma cells in the BM.

To test whether cf-mRNA profiling can be used to monitor the levels of the malignant Ig clone, we sequenced the cf-mRNA from plasma of these patients every day for 2 weeks after chemotherapy and transplant. While patient 1 showed no apparent reduction of the malignant clone after therapy (Supplementary Fig. 2D), patient 2 showed decreased levels of the predominant Ig variants in cf-mRNA after melphalan-induced apoptosis of plasma cells (Fig. 2b–d and Supplementary Fig. 2A–C). By day 10, the immune profile was no longer dominated by clonal Ig combinations, indicating successful therapy and BM reconstitution (Fig. 2b–d). In contrast, RNA-Seq performed on the matching buffy coat fraction throughout the study showed very limited information regarding the malignant Ig transcripts (Fig. 2c and Supplementary Fig 2A–C), supporting the potential of cf-mRNA to non-invasively capture BM activity.

### cf-mRNA reflects hematopoietic reconstitution after BM transplant

**Fig. 3:**
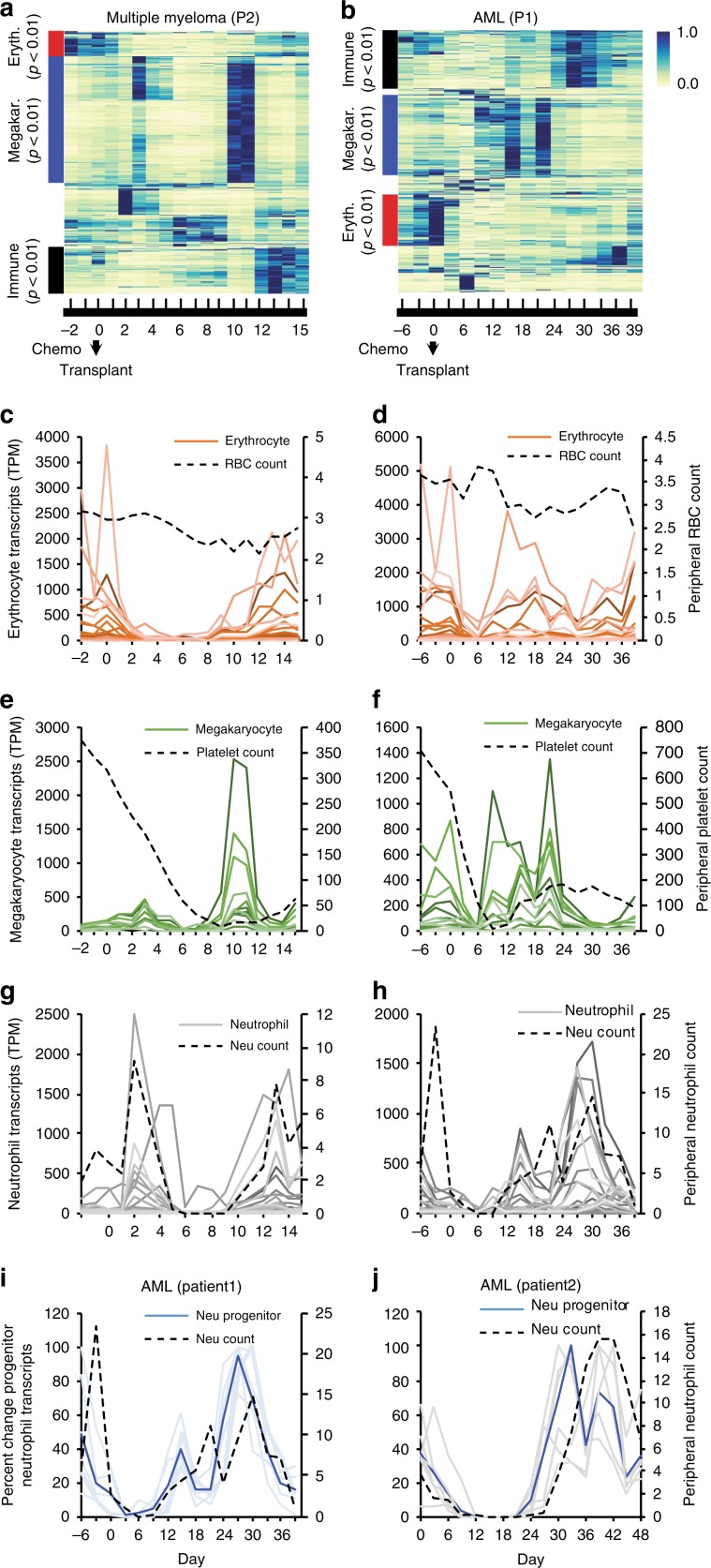
cf-mRNA reflects transcriptional activity of hematopoietic lineages during BM reconstitution. **a**, **b** Heat map of time-varying transcripts identified by cf-mRNA-Seq on multiple myeloma (MM) (**a**) and acute myeloid leukemia (AML) (**b**) patients undergoing BM ablation, followed by autologous or allogenic stem cell transplant, respectively (at day 0). Each column represents a time point with respect to the time of transplant, indicated in the bottom. Each row represents a gene. Enriched gene ontology terms for each cluster of transcripts are indicated (adjusted *p* value). **c**–**h** Time course of the levels of erythrocyte (red, **c**, **d**), megakaryocyte (green, **e**, **f**) and neutrophil (gray, **g**, **h**) specific transcripts in indicated MM (**c**, **e**, **g**) and AML (**d**, **e**, **h**) patients throughout the study. (Transcript identity is provided in Supplementary Table 5, detectable genes <5000 TPM used for visualization). Corresponding peripheral blood counts (every 3 days) are plotted in the secondary axis and represented with a black dotted line (RBC count, millions per mcL (**c**, **d**), platelet count, thousands per mcL (**e**, **f**) and neutrophil count, thousands per mcL (**g**, **h**). Day of blood collection with respect to transplant is indicated in the *X* axis. **i**, **j** Relative variation of progenitor neutrophil transcripts in AML patients 1 (**i**) and 2 (**j**) throughout the study. Average percent change for these transcripts is represented with a dashed blue lane. Dashed black line shows neutrophil counts in blood.

To gain further insight into the ability of circulating mRNA to reveal BM transcriptional activity, we followed the BM ablation and reconstitution dynamics after autologous HSC transplants in cf-mRNA, using the prototypical MM patient 2. Additionally, we investigated acute myeloid leukemia (AML) patients who underwent submyeloablative treatment followed by allogeneic HSC transplants (see Methods). Unsupervised clustering of transcripts detected in plasma cf-mRNA of MM and AML patients identified temporal patterns of expression for several groups of genes (Fig. 3a, b). Both Gene Ontology enrichment analysis and RNA-Seq data from Blueprint Consortium indicated that many of the identified components correspond to specific hematopoietic lineages (Fig. 3a, b). Therefore, we examined in detail the dynamics of hematopoietic lineage-specific transcripts (i.e., erythrocytes, megakaryocytes, neutrophils) in circulation during BM ablation and reconstitution.

First, to clarify the relationship between erythrocyte circulating transcripts and red blood cells (RBCs), we examined the levels of erythrocyte lineage-specific transcripts in plasma and RBC counts throughout the study. RBCs are the predominant cell type in circulation and are stable for ~120 days in the bloodstream^21^. Little variation in RBC numbers was noticed in MM and AML patients during the duration of these studies (Fig. 3c–d and Supplementary Fig. 4A). In contrast, most erythrocyte-specific transcripts in cf-mRNA were rapidly reduced after chemotherapy-mediated BM ablation in all patients, and recovered at later time points during BM reconstitution (Fig. 3c–d, Supplementary Fig. 3a–b, and Supplementary Fig. 4A). The discrepancy between RBC number and most erythrocyte transcripts in cf-mRNA indicates that these transcripts derive primarily from immature erythrocyte forms either in the BM or in circulation (reticulocytes), rather than from mature RBCs. We performed RNA-Seq analysis of paired buffy coat samples of MM patient 2 to gain further insight into the origin of these transcripts. The levels of erythrocyte-specific genes in CC are reduced after chemotherapy, resembling the dynamics observed in cf-mRNA (Supplementary Fig. 3C), and indicate that reticulocytes may be the source of most erythrocyte transcripts in WB. However, transcripts such as GATA1, a key transcriptional regulator of erythrocyte development, are detectable in cf-mRNA earlier than in buffy coat during BM reconstitution (Supplementary Fig. 3C), suggesting their BM origin. While future experiments will be necessary to discriminate the precise contribution of each compartment, our data show that erythrocyte transcripts derive primarily from immature erythrocyte cells residing in the BM and circulating reticulocytes, rather than from the highly abundant mature RBC.

To test whether discrepancies between cell blood counts (CBCs) and lineage-specific transcripts in circulation extend to other hematopoietic cell types, we next compared the dynamics of platelet counts and megakaryocyte-specific transcripts. In MM patient 2, a consistent increase in the levels megakaryocyte-specific transcripts is detected in cf-mRNA by days 9 and 10 after transplant, prior to platelet count recovery, which occurs by days 12 and 13 (Fig. 3e). Interestingly, RNA-Seq from matched buffy coat samples showed that megakaryocyte transcript levels in CC mimic the dynamic of platelet counts throughout the study (Supplementary Fig. 3c), and, unlike in cf-mRNA, early recovery of megakaryocyte transcripts is not detectable in CC during BM reconstitution. This disparity suggests that megakaryocyte transcripts detected in cf-mRNA during BM reconstitution, and before the platelet count recovers, are derived from the BM. Supporting this observation, in AML patient 1 megakaryocyte transcripts in circulation decreased after BM ablation and recovered by day ~9, clearly foreshadowing the increase in platelet counts occurring by days 12 and 13 (Fig. 3f). Strikingly, no recovery of this lineage occurred in cf-mRNA of AML patient 2 (Supplementary Fig. 4B). Follow-up BM biopsy confirmed lack of megakaryocyte development in this patient (Supplementary Table 9), demonstrating the specificity of the measured megakaryocyte signal. Thus, our data indicate that a fraction of megakaryocyte/platelet transcripts in circulation derive from the BM and that cf-mRNA reflects megakaryocyte transcriptional activity during BM reconstitution.

Last, we examined the kinetics of neutrophil counts and specific transcripts in circulation of MM and AML patients during the therapy. In MM patient 2, neutrophil counts showed two spikes, one shortly after transplant, likely due to the granulocyte-colony stimulating factor (G-CSF) treatment, which is followed by a rapid decrease due to BM ablation, and a second spike by days 12 and 13 likely indicative of BM reconstitution (Fig. 3g). This resembles the overall dynamics of neutrophil-specific genes in cf-mRNA and in buffy coat during the procedure (Fig. 3g and Supplementary Fig. 3E). However, while neutrophil transcripts in buffy coat and cf-mRNA peaked at a similar time to neutrophil counts during BM reconstitution, neutrophil precursor genes like CTSG increased earlier in cf-mRNA, by days ~8 and 9 after the autologous stem cell transplant. Supporting this observation, the levels of progenitor neutrophil transcripts in plasma of all AML patients decreased after BM ablation, and increased in cf-mRNA during BM reconstitution days earlier than the neutrophil counts (Fig. 3h–j and Supplementary Fig. 4D–F). These data further support that, during BM reconstitution, progenitor neutrophil transcripts in circulation are not derived from CC, but rather reflect BM transcriptional activity of the granulocyte lineage, providing valuable information about transplant engraftment and BM reconstitution.

**Fig. 4:**
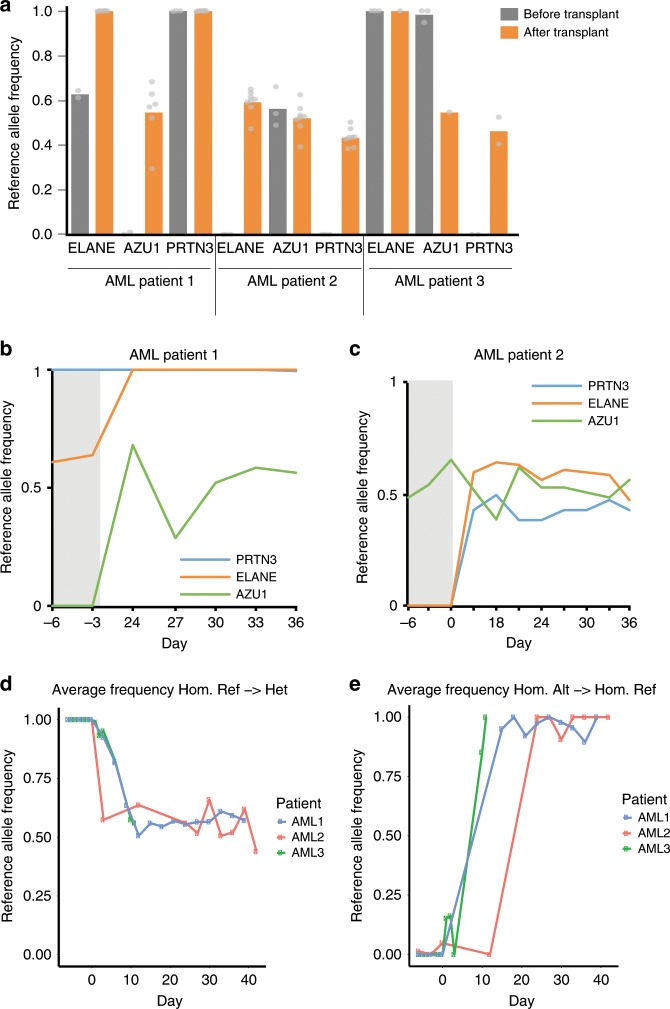
Monitoring of BM allotransplant engraftment in AML patients by cf-mRNA genotyping. **a**. Average frequency of reference allele of the SNPs detected in ELANE, AZU1 and PRTN3 neutrophil progenitor transcripts in cf-mRNA before (gray) and after (orange) allogeneic HSC transplantation in three AML patients, showing implantation of a new genetic profile after transplant. Source data are provided as a Source Data file. **b**, **c**. Frequency of reference allele of the SNPs detected in the same transcripts as in **a** for AML patients 1 and 2 over 36 days after transplant. Day of blood collection when SNPs were detected with respect to the time of transplant is indicated in the *X* axis. **d**, **e** Average reference allele frequency of all SNPs detected in the host cf-mRNA changing from reference homozygous to heterozygous (**d**) and from alternative homozygous to reference homozygous (**e**) after transplant. Day of blood collection is indicated in the *X* axis, transplant occurred at day 0.

We also investigated an orthogonal approach to measure transplant engraftment using cf-mRNA from AML patients receiving allogeneic HSC transplants, in which genetic differences exist between host and donor cells. Using a reference database of single-nucleotide polymorphisms (SNPs), we identified host-specific polymorphisms in progenitor neutrophil transcripts before the transplant (e.g., ELANE, AZU1, and PRTN3). After transplantation, these transcripts are substituted by new genetic variants from donor cells (Fig. 4a). Indeed, cf-mRNA profiling enabled monitoring of changes in these transcripts during therapeutic treatment of patients 1 and 2 (Fig. 4b–c). Combined analysis of all detected SNP from the host switching to a different genetic variant after transplant (e.g., from homozygous to heterozygous) indicates that multiple genetic differences can be identified in cf-mRNA to temporally monitor transplant engraftment (Fig. 4d–e). Altogether, our data show that cf-mRNA captures both genetic information and transcriptional activity from the BM, and enables monitoring of transplant engraftment and BM reconstitution from donor cells.

### cf-mRNA reveals response to stimulation with growth factors

**Fig. 5:**
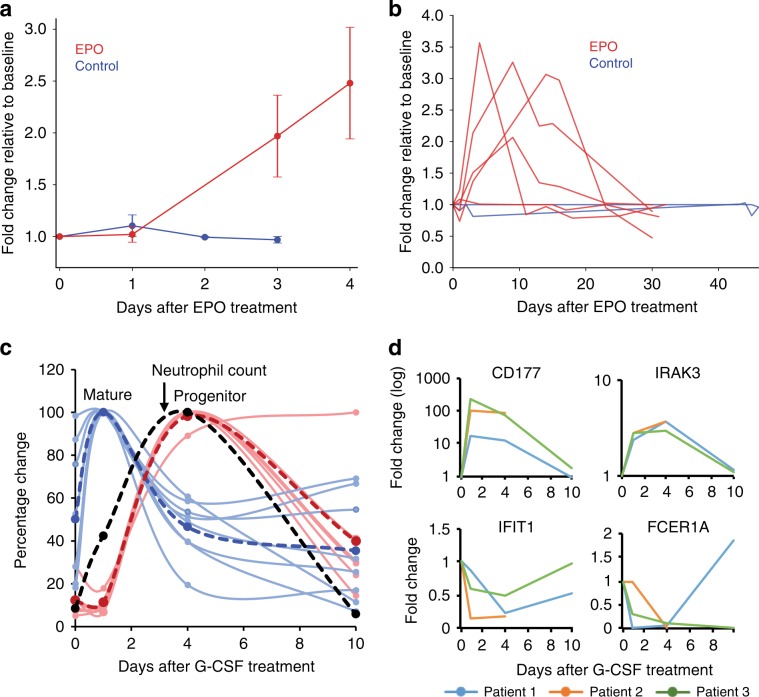
cf-mRNA captures the transcriptional activity of hematopoietic lineages upon stimulation. **a** Blood was obtained from nine patients before (day 0) and after being treated with a single EPO dose (see Methods). Gene expression patterns in plasma cf-mRNA were analyzed using RNA-Seq. Day 0 (before EPO treatment) was used as reference for each patient, and changes in the levels of erythrocyte-specific transcripts after EPO treatment calculated. Average fold change of erythrocyte transcripts in all *n* = 9 patients subjected to EPO treatment (red) and *n* = 3 untreated controls (blue) are shown. Error bars represent standard error (SE). Source data are provided as a Source Data file. **b** Time-course analysis of erythrocyte transcripts over a 30 day period in EPO-treated patients (red). Each line represents a patient, and shows average fold change of erythrocyte transcripts over time after a single EPO dosing administered at day 0, which is used as reference. Blue lines show fluctuations of the same transcripts in untreated healthy controls. **c** Blood was obtained from three healthy patients treated with G-CSF (before treatment (day 0), and 1, 4 and 10 days after treatment). Changes in circulating transcriptome were analyzed by cf-mRNA sequencing. Relative changes of immature (red) and mature (blue) neutrophil-specific transcripts throughout the study are shown for a representative patient treated with G-CSF. Dashed red and blue lines indicate the average for each group of transcripts. Relative changes in neutrophil counts are shown in black. Source data are provided as a Source Data file. **d** Time course of indicated G-CSF-responsive genes measured by cf-mRNA-Seq. Plots show fold change over time relative to day 0. Time points are connected by lines, each line represent a patient.

To evaluate the potential of cf-mRNA to monitor the activity of specific BM lineages after stimulation with growth factors, we obtained plasma from nine patients with varying degrees of chronic kidney failure on chronic maintenance erythropoietin (EPO) therapy. EPO is a peptide hormone that specifically increases the rate of maturation and proliferation of erythrocytes in the BM^22,23^. Samples were obtained prior to administration of EPO (day 0), and at several time points up to 30 days after treatment (see Methods). Average levels of erythrocyte transcripts across nine patients in cf-mRNA increased shortly after EPO treatment (Fig. 5a). The levels of erythrocyte transcripts continued to increase during the initial days after treatment compared to untreated control individuals (Fig. 5a, b). Indeed, key erythropoietic developmental transcripts involved in heme biosynthesis (e.g., ALAS2, HBB, HBA2) were induced in most patients (Supplementary Fig. 5A). Further, analysis of dysregulated genes (*p* < 0.05) in plasma at days 3 or 4 after treatment with EPO using IPA (Ingenuity Pathway Analysis) showed “Heme biosynthesis II” as the top enriched pathway (*p* < 0.001), supporting the transcriptional induction of this cell lineage. Thirty days after EPO treatment, erythrocyte transcripts returned to basal expression levels in these patients (Fig. 5b and Supplementary Fig. 5). Thus, our longitudinal studies indicate that cf-mRNA levels reflect specific transient stimulation of the erythroid cell line.

As another approach to study in vivo changes in cf-mRNA upon perturbation of a cell lineage, we collected samples from three healthy patients who received G-CSF treatment, a well-known pro-survival factor for neutrophilic granulocytes. Blood was drawn before the treatment and at 1, 4, and 10 days after G-CSF stimulation (the 10-day time point and CBC could only be obtained for two patients, see Methods). As expected, neutrophil count increased after G-CSF treatment, peaking at day 4, and returned to basal levels by day 10 (Fig. 5c). Neutrophil-specific transcripts in plasma cf-mRNA showed a bimodal increase after G-CSF treatment for all patients (Fig. 5c and Supplementary Fig. 5B, C). Neutrophil progenitor-specific transcripts increased in cf-mRNA coinciding with the peak in neutrophil counts likely as a consequence of G-CSF-mediated mobilization of granulocytes from the BM into circulation (Fig. 5c and Supplementary Fig. 5B). However, mature neutrophil transcripts rapidly increase in cf-mRNA one day after the treatment, foreshadowing the peak of neutrophil counts (Fig. 5c and Supplementary Fig. 5C). This suggests a direct and transient transcriptional response of neutrophils to G-CSF. Indeed, transcripts previously reported both in vivo and in vitro to increase (e.g., IRAK3) or decrease (e.g., IFIT1) in neutrophils in response to G-CSF, followed the expected trend^24^ (Fig. 5d). Altogether, our results indicate that cf-mRNA reflects dynamic cell-type-specific transcriptional responses to stimulation.

## Discussion

The growing interest to identify non-invasive alternatives to standard tissue biopsies has generated enormous attention for liquid biopsies over the last decade. Initial advances in cfDNA technology have paved the way for the development of clinically applicable cf-NA-based biomarkers^25–27^. cfDNA offers potential advantages compared to invasive tissue biopsies; however, cfDNA analyses largely rely on mutations, polymorphisms, or structural variations, compromising its use in disease and physiological scenarios not associated with genetic differences. To partially circumvent these limitations, cfDNA methylation analyses have recently been used as a proxy of tissue-specific gene expression, but further work is needed to validate this approach^28^. For RNA-based non-invasive biomarkers, non-coding RNAs including miRNA and lncRNA have been studied extensively in multiple diseases^29^. While these developments in cell-free RNA are intriguing, functional annotation of non-coding RNAs remains poorly characterized. In contrast, the cf-mRNA transcriptome provides direct access to both genetic information and information pertaining to the tissue of origin and its physiology. For instance, we have shown that genetic alterations in cf-mRNA provide valuable information for monitoring allografts, and similar approaches have shown their value in diagnosing fetal chromosomal abnormalities^30^. Given that several studies have identified tumor-derived transcripts in the circulation^14^, the genetic information captured by cf-mRNA is of particular interest in cancer diagnosis and monitoring. In addition, cf-mRNA provides tissue-specific transcripts that reveal functional information pertaining to the tissue of origin. While further experiments in larger cohorts will be necessary to determine the clinical utility of cf-mRNA, we showed that cf-mRNA captures transcripts that reveal BM physiology. Similarly, previous studies have reported transcripts in circulation encoding functional information of the liver, brain, immune system, or fetal development^7,31,32^. Therefore, cf-mRNA has the capability of integrating functional and genetic information of tissues, highlighting this analyte’s unique potential as a non-invasive biomarker.

Another key aspect of non-invasive approaches is that by eliminating the need for surgical tissue acquisition they enable repeated, longitudinal assessment of a patient’s disease state over time. This could be particularly relevant in clinical settings, such as monitoring of treatment in cancer patients, where biopsy of affected tissue remains the accepted reference method. In this regard, our longitudinal cf-mRNA profiling data provides evidence for circulating transcript snapshots of gene expression profiles in tissues such as BM. Longitudinal cf-mRNA monitoring may allow non-invasive temporal delineation of BM ablation efficiency, early detection of transplant engraftment, and monitoring of BM reconstitution. For example, in MM patients, cf-mRNA profiling integrates temporal measurement of clonal Ig transcripts generated by malignant plasma cells in the BM, with detailed BM-lineage transcriptional activity and establishment of a new immune profile. The comprehensive picture revealed by cf-mRNA profiling provides additional relevant information compared to other non-invasive tests commonly used in this malignancy, such as clonal antibody detection in serum of MM patients. Indeed, given the challenging and subjective quantification and characterization of these antibodies, BM biopsies remain a common practice in the therapy management of MM patients^33^. In addition, unlike antibody detection, cf-mRNA profiling has the potential for early identification of suboptimal BM reconstitution, as shown by the lack of development of megakaryocyte lineage in AML patient 2. While our study is based on a limited number of patients, our data provide promising initial proof of concept of using cf-mRNA profiling to monitor BM activity, which could lead to improved therapeutic management of patients with BM disease, and eventually alleviate the need for invasive BM biopsies.

Finally, understanding the mechanisms underlying the presence of mRNA transcripts in circulation is essential to interpret their clinical value. For example, cfDNA is expected to originate primarily from dying cells^13^; therefore, the use of this liquid biopsy likely relies on scenarios associated with cell death. While further experiments will be necessary to formally rule out the hypothesis of cell turn over as the exclusive source of cf-mRNA in vivo, our data suggest that changes in cf-mRNA levels are influenced by transcriptional changes in living cells during maturation, proliferation, and response to stimuli, without requiring cell death. For example, we showed that melphalan-induced apoptosis did not significantly increase the levels of cf-mRNA. In contrast, a large increase of transcripts in circulation was observed during BM reconstitution and upon stimulation with well-known pro-survival and antiapoptotic growth factors. Supporting our interpretation, in vitro studies indicate that extracellular mRNA levels and composition change upon cellular stimulation^34,35^ and that living cells can secrete RNA molecules encapsulated in vesicles. Additionally, our longitudinal clinical studies demonstrate that the circulating transcriptome is a dynamic metric that allows constant measurement of tissue function over time. Alternatively, cfDNA methylation and mutation events are less dynamic and likely provide limited information on tissue homeostasis and disruption. In summary, cf-mRNA profiling may provide richer molecular content compared to other non-invasive biomarkers and constitutes a unique non-invasive interrogation of tissue function in scenarios such as monitoring of disease and drug engagement and response in patients.

## Methods

### Samples and patients

MM patients eligible for autologous marrow transplantation were recruited from the Scripps Bone Marrrow Transplant Center. Patients with non-secretory disease or plasma cell leukemia were excluded. Three total patients were enrolled with daily blood draws collected throughout the cytoreductive conditioning regiment and subsequent hospital stay. High-dose melphalan was used to ablate the marrow over a 2-day conditioning regiment, followed by transplantation of HSCs. Sequential daily collections discontinued the day of hospital discharge. Follow-up BM biopsy occurred between 60 and 90 days. Complete blood counts (CBCs) were collected as a part of the study. Plasma was processed within 2 h of blood collection and stored at −80 °C. Patient characteristics are described in Supplementary Table 6.

EPO-treated patients were recruited for study enrollment provided they were administered erythropoietin as part of routine medical care. Potential patients were excluded if they were (1) currently on any anti-cancer therapy; (2) had active hemolysis from any cause, or (3) were pregnant. Patients were consented and enrolled from the Renal and Hematology/Oncology Clinics at Scripps Clinic Cancer Center. Per standard clinical care, a single dose of EPO was administered per month. Blood was collected at day 0 (before administration of EPO), and at days 1, 4, and 10 after administration of EPO. Days 4 and 10 collections were allowed for ±1 day adjustment to accommodate patients’ schedules. A subset of patients consented to an expanded protocol allowing for blood collections up to day 30. CBCs were performed as well. Plasma was processed within 2 h of blood collection and stored at −80 °C for batch processing. Patient characteristics are shown in Supplementary Table 7.

Specimens from healthy controls were obtained from the San Diego Blood Bank, processed, frozen, and stored at −80 °C for batch processing.

G-CSF patients, normal healthy individuals preparing to donate peripherally harvested stem cells for allo-transplants, were recruited from Scripps and enrolled as part of our G-CSF cohort. In total, three patients were consented and donated blood during their stem cell mobilization. Patient characteristics are shown in Supplementary Table 8. Blood was collected at day 0 (before administration of G-CSF), and at days 1, 4, and 10 after administration of G-CSF. Day 4 and 10 collections were allowed for ±1 day adjustment to accommodate patients’ schedules and additionally, the day 10 collection was optional. CBCs were performed for each sample. Samples were processed within 2 h of blood collection and stored at −80 °C for batch processing.

Patients with known AML, in preparation for submyeloablative treatment and allogeneic stem cell transplantation as part of standard care, were recruited for daily blood draws throughout their treatment and stem cell transplant. Three patients were enrolled in our study (characteristics in Supplementary Table 9), and submyeloablative treatment were generally 6 days, using a combination of fludarabine and melphalan to obtain a partial ablation of the marrow, prior to transplantation. HSCs obtained from a single donor, were administered on day 0, and blood draws were continued through the hospital stay. Patient 3 was discharged by day ~15 and in-hospital collections were limited to day 45 post transplant. Follow-up routine BM biopsies were performed. CBCs were collected. cf-mRNA was sequenced every 3 days. Plasma was processed within 2 h of blood collection and stored for batch processing.

### Patient consent

All studies were approved by their respective institutional IRBs and patients consented according to submitted study protocols. We have complied with all relevant ethical regulations. Molecular Stethoscope maintained approval for blood collection and research through Western IRB Protocol #20162748, under which healthy control samples were collected. In collaboration with the Scripps Cancer Center and the Blood & Marrow Transplant Program at Scripps Green Hospital, G-CSF and EPO studies were conducted under Scripps Institutional Review Board-approved protocol IRB-16-6808. Our studies involving hematopoietic BM transplants, for both MM and AML, were approved by and conducted in accordance with Scripps IRB Protocol IRB-17-6953, in collaboration with the same groups.

### Sample processing

Blood samples were collected in EDTA tubes (BD #366643) for plasma processing or in BD Vacutainer red-top clotting tubes (BD #367820) for serum processing. The biofluid used in each experiment is indicated in the main text as well in the corresponding cohort details in this section. Blood samples were kept at room temperature and samples were processed within 2 h after blood draw. Plasma and serum volume ranging from 500 μl to 1 ml was used for the extractions. Samples were first centrifuged at 1900 × *g* for 10 min. Plasma and serum were separated into new tubes. To remove cell debris, we subsequently centrifuged serum/plasma at 16,000 × *g*. For cancer patient plasma samples (MM and AML), the second centrifugation step was performed at 6000 × *g*. Plasma/serum samples were immediately frozen and stored at −80 °C. Freeze/thaw cycles were avoided. Buffy coat samples were obtained by isolating the buffy coat layer enriched in white blood cells after initial centrifugation of blood samples. Nucleic acids were isolated from plasma/serum using the Circulating Nucleic Acid Kit (Qiagen). ERCC RNA Spike-In Mix (Thermo Fisher Scientific, Cat. #4456740) was added during the extraction process as an exogenous spike-in control according to manufacturer’s instruction (Ambion). Nucleic acids from WB and buffy coat samples were extracted with TRIzol LS (Thermo Fisher) following the manufacturer’s instructions. Subsequently, RNA and cf-RNA samples were incubated for 25 min with 3 μl of the inhibitor resistant rDNase (Turbo DNase, Invitrogen) to eliminate any remnant DNA and concentrated afterwards. RNA was eluted in 15 μl of RNase free water. The amount, size, and integrity of cf-RNA was estimated by running 1 μl of the sample in an Agilent RNA 6000 Pico chip using a 2100 Bioanalyzer (Agilent Technologies) and confirmed by quantitative PCR (qPCR). Twenty-five to thirty percent of the cf-RNA eluate was converted to cDNA using random hexamers, NGS libraries were generated and whole-exome was captured prior to Illumina sequencing. Libraries were quantified by qPCR with Kapa Quantification Kit (Kapa) and in a Quantifluor (Agilent Quantus Fluorometer, Promega) using QuantiFluor ONE dsDNA Kit (Promega), and library size was checked on the Bioanalyzer (Agilent Technologies) using high-sensitivity DNA chips (Agilent Technologies). Samples were pooled and sequenced on a NextSeq 500 (Illumina) platform according to the manufacturer’s instructions.

### Sequence data processing, alignment, and quantification

Base calling was performed on an Illumina BaseSpace platform, using the FASTQ Generation Application. Adaptor sequences are removed and low-quality bases trimmed, using cutadapt (v1.11). Reads shorter than 15 base pairs were excluded from subsequent analysis. Read sequences are then aligned to the human reference genome GRCh38 using STAR (v2.5.2b) with GENCODE version 24 gene models. Duplicated reads are removed by invoking the samtools (v1.3.1) rmdup command. Gene expression levels were inferred from de-duplicated BAM files using RSEM (v1.3.0).

### Differential expression analysis

Differential expression analysis between different conditions was performed using DESeq2 (v1.12.4). RSEM-estimated read counts are used as input for DESeq2. Genes with fewer than 20 reads across the samples are excluded from this analysis. Potential Gene Ontology enrichment and involvement on biological pathways of genes were examined using the R package limma (v3.28.21) and IPA software (Qiagen)

### Cell-type-specific genes

Tissue (cell-type)-specific genes are defined as genes that show much higher expression in a particular tissue (cell type) compared to other tissues (cell types). Information about tissue (cell-type) transcriptome expression levels was obtained from the following two public databases: GTEx^36^ for gene expression across 51 human tissues and Blueprint Epigenome^37^ for gene expression across 56 human hematopoietic cell types. For each gene, the tissues (cell types) were ranked by their expression of that particular gene, and if the expression in the top tissue (cell type) is >20-fold higher than all the other tissues (cell types), the gene was considered specific to the top tissue (cell type). For the establishment of BM-enriched transcripts, we performed RNA-Seq on a commercial human BM total RNA sample. Subsequently, BM transcriptome was compared to WB transcriptome to identify genes enriched in BM and WB transcriptomes (fold change >5).

### Immunoglobulin gene repertoire in MM patients

For clone-type assembly, we performed de novo transcriptome assembly using Trinity. Next, the assembled contigs were compared to Ig gene annotation database IMGT^38^ using igBLAST (v2.5.1) to identify the V(D)J combinations. To quantify the relative abundance of variable region genes, we collected reads that were either unaligned to the human reference genome or aligned to an annotated Ig gene by STAR and add them to the sequences in the IMGT database using igBLAST. Relative abundance was calculated as the ratio of the number of reads mapped to a particular Ig gene over the total number of reads mapped to any Ig gene.

### Unsupervised clustering

Genes that met the following two criteria were selected for clustering: (1) the maximum expression across time points higher than 50 TPM; (2) the ratio of the highest expression over the lowest was >5. For each of the selected genes, the expression values were normalized by dividing each value by the maximum value across all time points. The purpose of this normalization was to bring all the genes to a comparable scale and focus on their relative changes across time points instead of their absolute expression levels. *K*-means and hierarchical clustering were then performed to find genes that share similar temporal expression patterns.

### Non-negative matrix factorization

Genes whose expression was lower than 20 TPM in all samples were excluded from the decomposition analysis. For each of the remaining genes, the expression values were normalized by dividing each value by the maximum value across all samples. The purpose of this normalization step is to bring all the genes to a comparable scale. NMF was then performed on the normalized values to decompose the genes into 8–12 components. NMF decomposition was implemented by invoking the “decomposition.NMF” class in the sciki-learn Python library. NMF decomposition creates groups of genes (components) sharing similar expression patterns (correlated across samples) in an unsupervised manner, thereby revealing underlying structures within the data. In order to better annotate the discovered components, we selected genes enriched in a particular component (i.e., those genes that have the highest loadings within the component) and examined (1) their expression levels across 51 human tissues in GTEx; (2) their expression levels across 55 human hematopoietic cell types from the Blueprint Epigenome consortium; (3) their Gene Ontology functional enrichment. If most of these genes show high expression in a certain cell type (e.g., platelet) or are enriched in certain biological processes (e.g., platelet activation and coagulation), we will designate the component accordingly (e.g., megakaryocyte component). By integrating those three sources of information, we are able ascertain the tissue/cell-type origin for most components.

### Reporting summary

Further information on research design is available in the Nature Research Reporting Summary linked to this article.

## Supplementary information

Supplementary Information

Reporting Summary

## Data Availability

RNA-Seq datasets have been deposited online in Sequence Read Archive (SRA) under accession numbers PRJNA517339. Source data underlying Figs. 1B–F, 4A, 5A, C and Supplementary Figs. 1D, E are provided as a Source Data file.

## Source data

Source Data

## Code availability

Custom code used during the current study are also available at Bitbucket https://MS_JialiZhuang@bitbucket.org/MS_JialiZhuang/naturecomm2019-related-codes.git.
