# Supplementary material for: Non-invasive characterization of human bone marrow stimulation and reconstitution by cell-free messenger RNA sequencing
Source: Nat Commun. 2020 Jan 21;11:400. doi: 10.1038/s41467-019-14253-4 (PMC6972916; doi:10.1038/s41467-019-14253-4)
Supplement: Supplementary file 1 — Supplementary Information [file 41467_2019_14253_MOESM1_ESM.pdf]

Supplemental Information

Non-invasive characterization of human bone marrow stimulation and reconstitution by cell-free messenger RNA sequencing

Ibarra et al.

Supplementary Figure 1

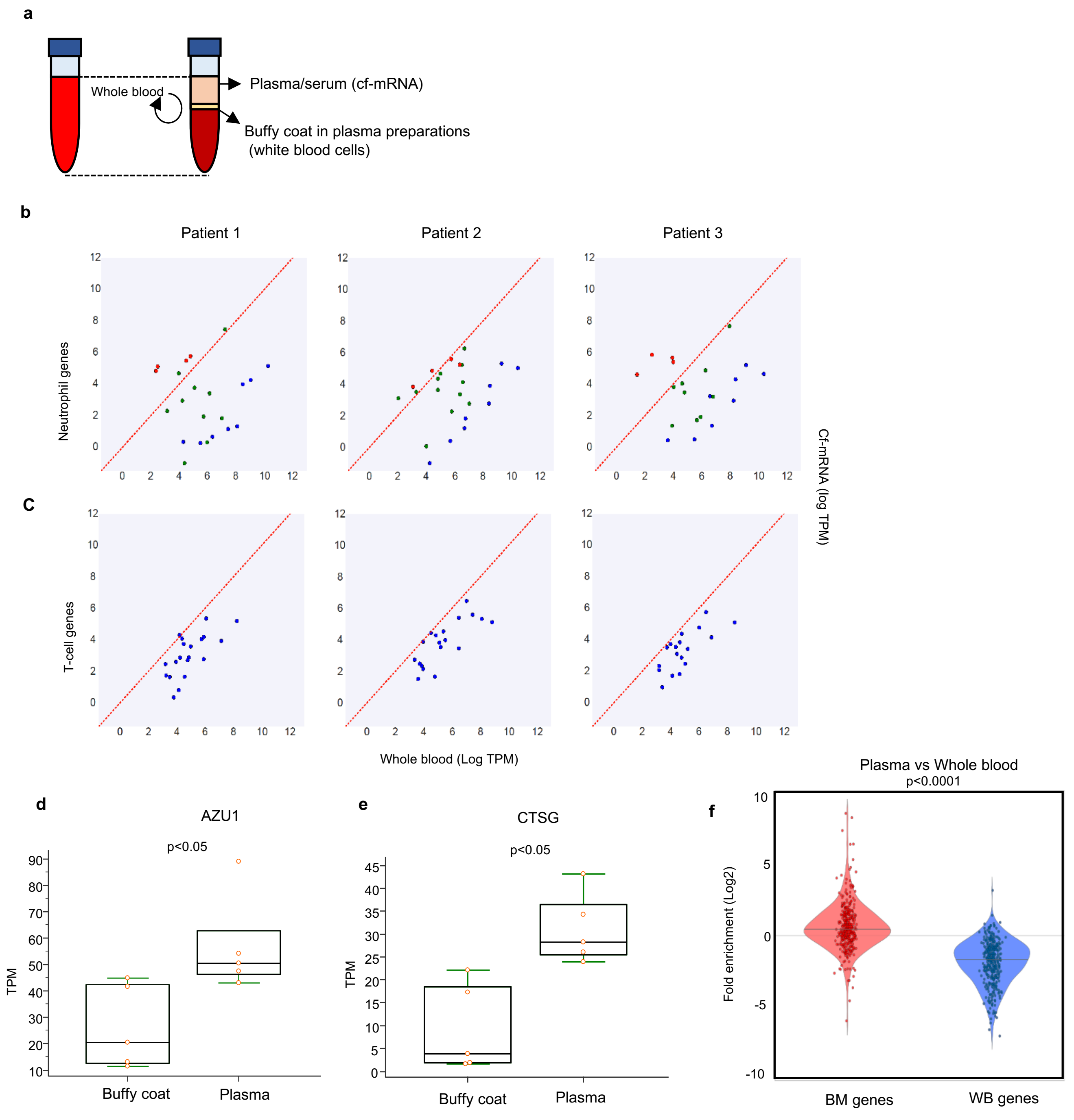

Supplementary Figure 1. cf-mRNA transcriptome is enriched in bone marrow transcripts compared to circulating cell transcriptome. a, Schematic of whole blood, plasma and buffy coat collection. **b-c**, Scatter plots comparing the levels in peripheral blood (X axis) and cf-mRNA (Y axis) of neutrophil-specific and T-cell-specific transcripts. Neutrophil progenitor transcripts are shown in red, mature transcripts in blue, general neutrophilic transcripts in green. Both x-axis and y-axis show TPM in $\log_2$ scale. See also Supplemental Table 5. **d-e**, Box-plots comparing the normalized levels (TPM) of the indicated hematopoietic progenitor transcripts measured by RNA-Seq in paired buffy coat and cf-mRNA samples (n=5; p-values d) 0.016, e) 0.009, U d) -2.40, e) -2.61, two-sided Wilcoxon ranked sum test). Center line, median; box limits, upper and lower quartiles; whiskers, 1.5x interquartile range; points, outliers. Source data are provided as Source Data file. **f**, Levels of BM-specific (red dots) and whole blood WB-specific genes (blue dots) were compared in matching plasma and whole blood of 3 individuals. Average fold change (plasma/whole blood) of these transcripts is shown, p value < 0.0001, Wilcoxon rank-sum test.

Supplementary Figure 2

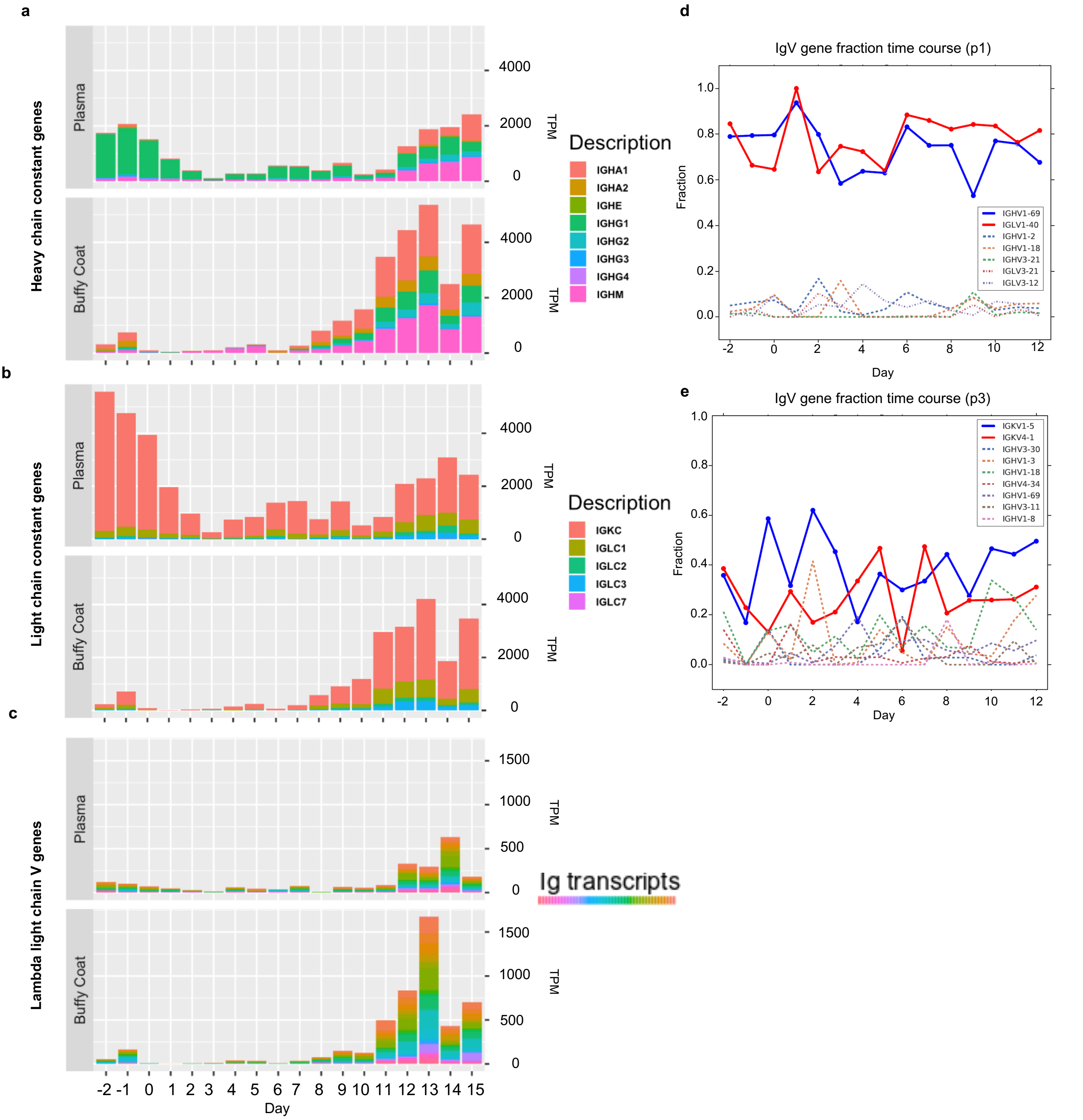

Supplementary Figure 2. cf-mRNA contains Ig transcripts derived from plasma cells in the BM of Multiple Myeloma patients. a-c, Levels of Ig transcripts measured by RNA-Seq in plasma and buffy coat of multiple myeloma (MM) patient 2, undergoing BM ablation and autologous stem cell transplantation (day 0). Bar graphs show the normalized levels (TPM) of Ig heavy chain constant region transcripts (**a**), light chain constant region transcripts (**b**) and lambda light chain variable region transcripts (**c**) detected during the study. Day of blood collection with respect to the time of transplant is indicated in the X axis. **d-e**, Fraction of Ig heavy and light variable chain transcripts over time in cf-mRNA of MM Patient 1 (d) and Patient 3 (e). Dominant transcripts are shown in solid blue and red lines. Time with respect to transplant day is shown.

Supplementary Figure 3

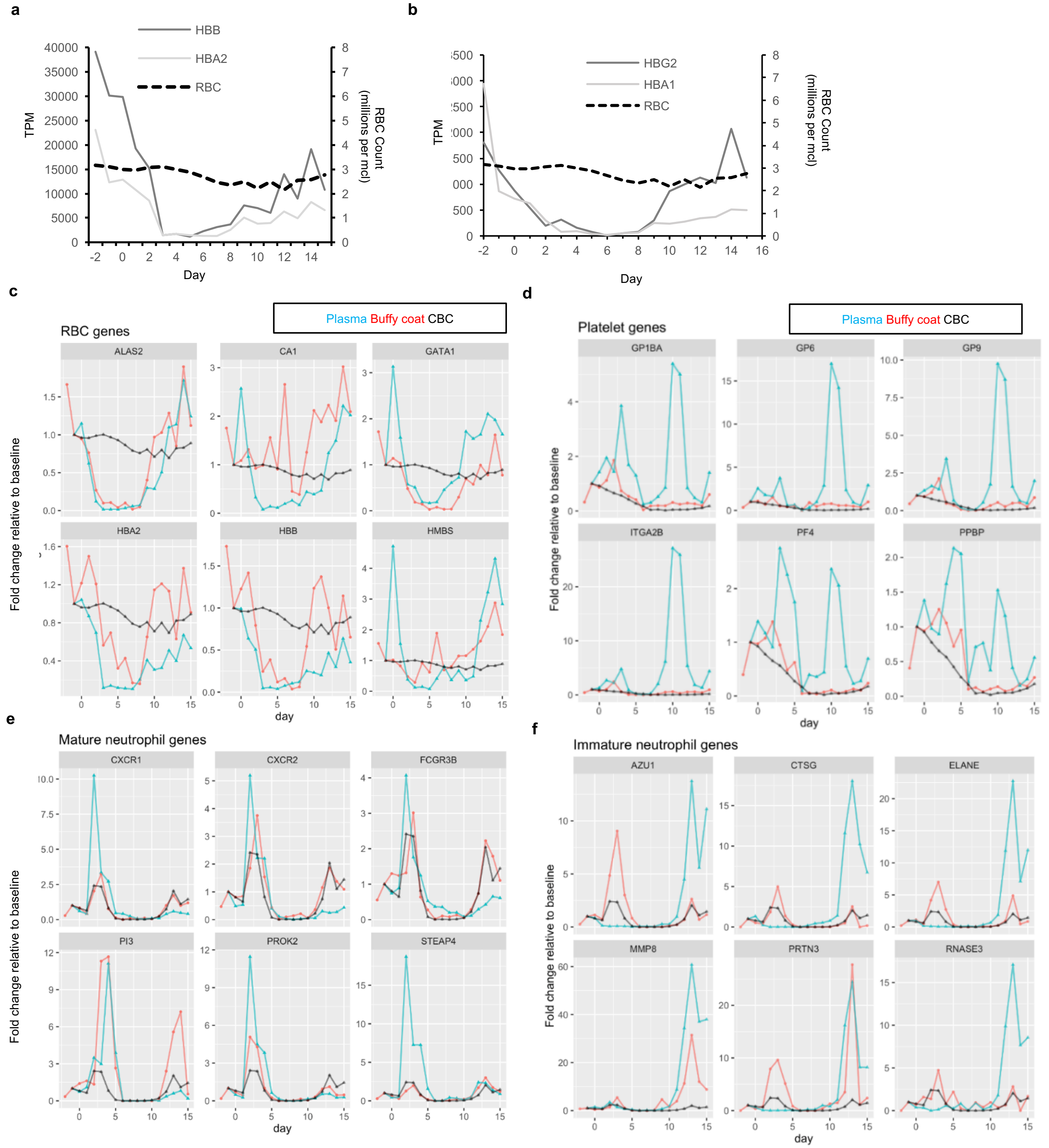

Supplementary Figure 3. Monitoring BM transcriptional activity by cf-mRNA profiling in a Multiple Myeloma patient during BM ablation and transplant. a-b, Time course of red blood cell counts (RBC, dashed black line) and indicated hemoglobin transcripts (grey) in multiple myeloma Patient 2 during chemotherapy and BM reconstitution. Day of blood collection with respect to the time of transplant is indicated in the X axis. **c-f**, RNA-Seq was performed in cf-mRNA (blue) and matching buffy coat samples (red). Graphs show the fold change relative to baseline of indicated erythrocyte (**c**) and megakaryocyte (**d**), mature neutrophil (**e**) and immature neutrophil transcripts (**f**) in both specimens. In all panels, black lines represent the relative changes in corresponding circulating cell blood counts: RBC counts (**c**), platelet counts (**d**) and neutrophil counts (**e**, **f**). Day of blood collection with respect to the time of transplant is indicated in the X axis.

Supplementary Figure 4

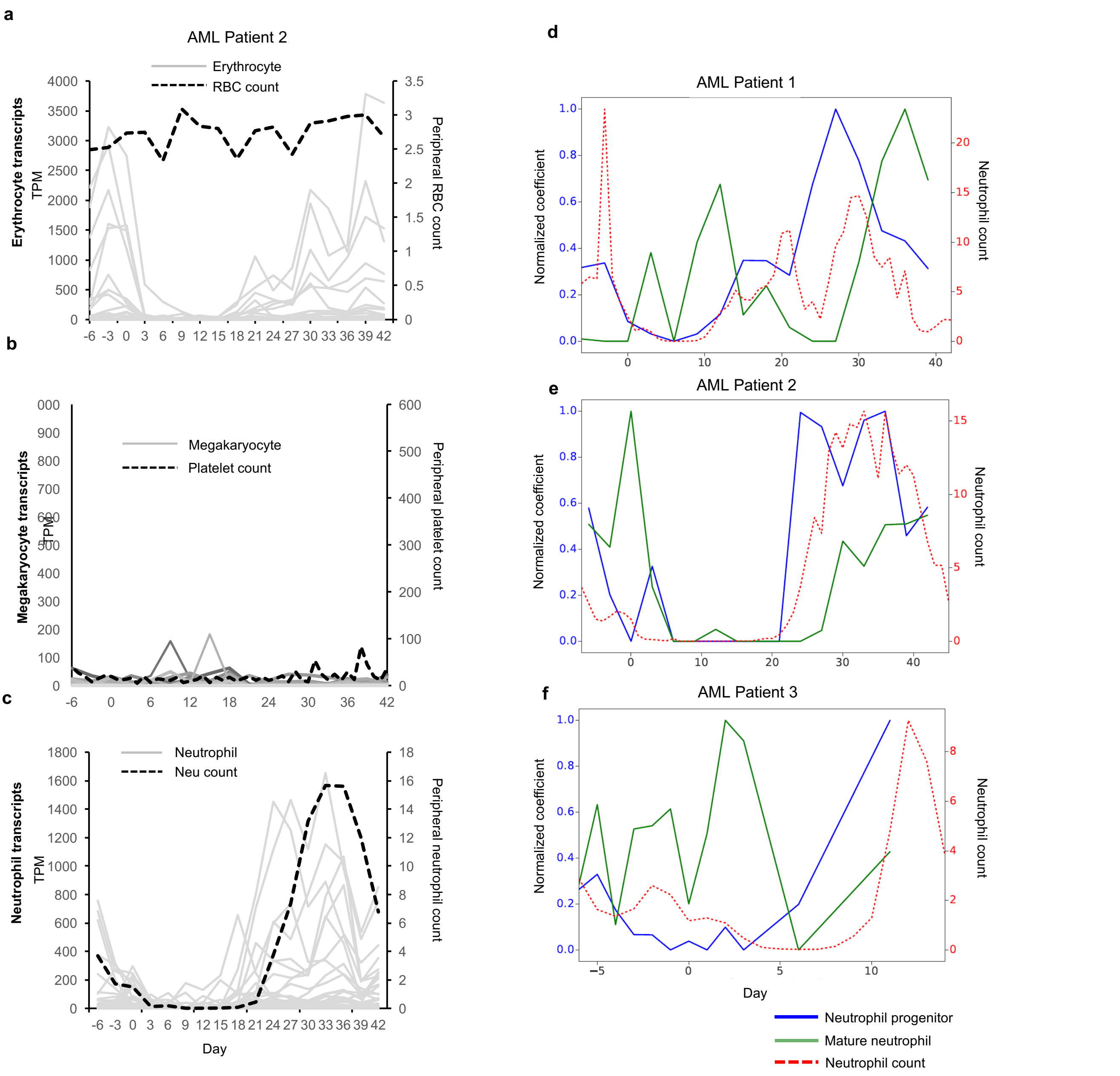

Supplementary Figure 4. Monitoring transcriptional activity of BM hematopoietic lineages by cf-mRNA in Acute Myeloid Leukemia (AML) patients undergoing BM transplant. a-c, Time course of normalized levels (TPM) of erythrocyte (**a**), megakaryocyte (**b**) and neutrophil (**c**) specific transcripts in AML Patient 2 (grey lines). Corresponding peripheral blood counts (every 3 days) are plotted in the secondary axis of each graph and represented with a black dashed line (RBC count M/mcl (**a**), platelet count K/mcL (**b**) and neutrophil count K/mcl (**c**)). Day of blood collection with respect to the time of transplant (day 0) is indicated in the X axis. Cell-type specific transcripts can be found in Supplementary Table 5, genes <5,000 TPM were used for visualization, and <2,000 TPM for panel c. **d-f**, Time course of mature (green) and immature neutrophil NMF components (blue) in AML patients 1 (d), 2 (e) and 3 (f). Daily neutrophil count is plotted in red. Day of blood collection with respect to the time of transplant is indicated in the X axis.

Supplementary Figure 5

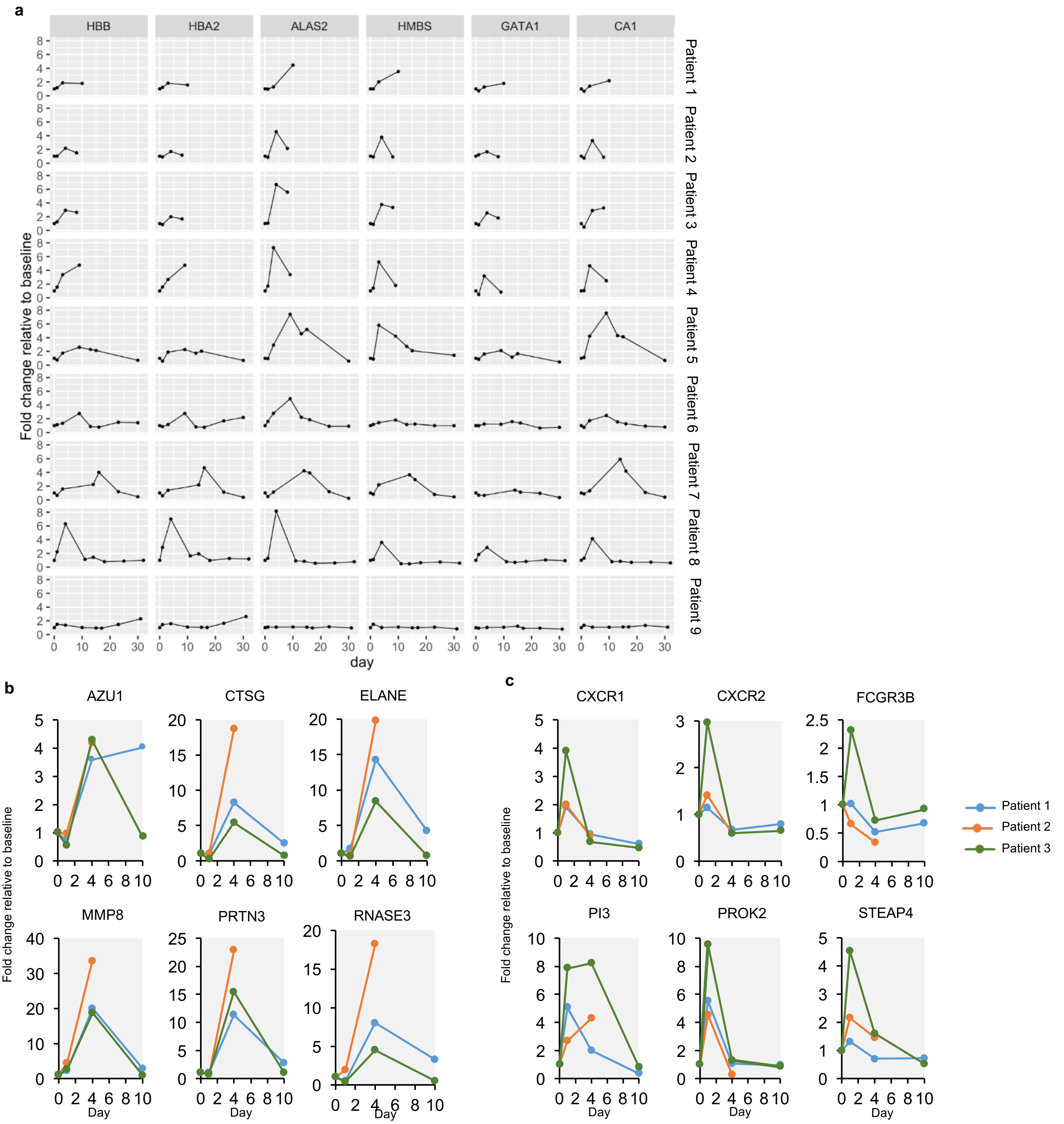

Supplementary Figure 5. Induction of lineage specific-genes in cf-mRNA by growth factors. a, Fold change over time of indicated erythrocyte genes (top) in plasma of EPO treated patients (right) relative to baseline. **b-c**, Fold change of immature (**b**) and mature (**c**) neutrophil specific transcripts in cf-mRNA of a patients after treatment with G-CSF. Day 0 (before treatment) is used as reference. Fold change of indicated transcripts is shown for 3 patients, represented in different colors. Time points across each Patient are connected by lines. Day of blood collection with respect to the time of treatment is indicated in the X axis.

Supplementary Table 1. Average number of transcripts detected in cf-mRNA of healthy donors (n=24)

TPM Criteria	>40% of the samples	>60% of the samples	>80% of the samples
TPM > 1	12341	11393	10313
TPM > 5	9414	8485	7334

TPM (Transcripts Per Million)

Supplementary Table 2: Summary of sequencing metrics

Sample ID	Reads aligned to mRNA (%)	Reads aligned to intron (%)	Duplication rate	Correlation with ERCC (PCC)	Unique fragment	Protein coding genes detected*
12687-A1	64.9	7.7	10.5	0.95	306643	10183
12687-A2	70.6	5.6	7.4	0.97	264871	9718
12819-A1	87.2	1.2	13.3	0.94	314330	9652
12819-A2	89.5	1.8	13.5	0.93	397425	10204
12824-A1	82.8	3.5	14.3	0.96	552282	11007
12824-A2	91.9	1.2	9.2	0.93	583604	11106
12829-A1	90.0	1.5	12	0.96	473651	10561
12829-A2	90.5	1.2	11.4	0.89	492788	10691
12835-A1	94.5	1.1	11.9	0.96	861572	12118
12835-A2	89.0	1.9	10.1	0.95	757347	12028
12841-A1	87.2	2.6	17.6	0.91	524589	10742
12841-A2	94.3	1.1	10.2	0.98	774486	11587
12846-A1	90.1	1.2	16.2	0.92	591508	11196
12846-A2	93.7	1.2	12.2	0.93	604647	11248
12852-A1	90.5	1.9	11.7	0.89	433837	10251
12852-A2	90.7	1.8	7.4	0.88	412466	10168
12858-A1	89.9	2.3	24	0.93	839497	11886
12858-A2	91.3	1.8	20.9	0.92	676180	11351
12864-A1	88.7	2.3	8	0.97	474861	10933
12864-A2	88.9	2.3	5.1	0.97	442572	10784
13079-A1	84.5	3.3	4.6	0.97	474443	10455
13079-A2	84.8	3.3	3.2	0.91	422299	10224
13086-A1	89.9	2.1	5.9	0.97	657814	11390
13086-A2	90.1	2.1	3.8	0.96	593309	11221
13092-A1	85.9	1.2	14	0.96	605880	11036
13092-A2	89.2	1.5	8.7	0.91	376971	10101
13096-A1	88.5	2.0	13.6	0.93	311271	9952
13096-A2	88.6	2.0	8.5	0.93	298347	9799
13103-A1	76.2	5.0	13.5	0.96	471299	10361
13103-A2	80.0	3.7	13.5	0.95	366955	9803
13110-A1	78.3	4.7	4.2	0.95	1520926	12952
13110-A2	91.2	2.1	3.2	0.88	1792888	13193
13120-A1	78.6	4.3	8.9	0.96	399780	9493
13120-A2	81.4	1.3	12.6	0.95	492775	9751
13126-A1	92.0	1.1	20.9	0.96	444705	10655
13126-A2	91.4	1.0	19.9	0.92	435998	10760
13129-A1	71.3	6.4	6	0.96	478551	10784
13129-A2	88.3	2.4	5	0.95	656115	11371
13136-A1	85.2	1.4	8.2	0.95	510213	10924
13136-A2	85.0	2.6	6	0.94	581233	11260
4510-A1	73.4	2.8	6.6	0.92	738901	12253
4510-A2	67.2	1.2	12	0.96	328331	10189
9709-A1	91.0	1.0	8.6	0.93	991082	12406
9709-A2	81.0	3.3	8.7	0.95	827893	12377
9737-A1	90.8	0.7	6.3	0.96	1331072	12857
9760-A1	87.4	1.0	15.1	0.91	828881	12256
9760-A2	78.1	3.0	14.4	0.96	468786	11064

***TPM is greater than equal to 2. A1 and A2 denote replicates. PCC: Pearson's correlation coefficient**

Supplementary Table 3: List of bone marrow enriched genes compared to whole blood

Gene ID						
PRTN3	BEX1	MYL2	ARHGAP11A	SLC15A2	BGN	HMGB3
CTSG	HJURP	HSPA1B	NXF3	ENTPD7	FOLR3	ZWINT
ELANE	HIST1H2AJ	HIST1H4I	ARG1	IQGAP3	SLC1A3	TUBB
MPO	NUCB2	S100A9	CP	WDR34	RRM1	RP11-65L3.2
DEFA4	MMP9	MB	SYNGR1	MGST1	APOA1	RAD54L
MMP8	CAMP	PLTP	GGH	HIST2H2AB	SMC2	TTN-AS1
CD177	PLK1	UGCG	PCOLCE2	FAM178B	TUBG1	WEE1
CXCL12	PGLYRP1	HIST1H2AI	BCL2L15	FUT4	DLC1	EPAS1
OLFM4	CRISP3	VCAM1	DES	LBP	HIST1H2BJ	PIF1
AZU1	SEPP1	MLC1	PSAT1	ITGA9	HELLS	INCENP
DEFA3	C1QB	HIST1H3I	CPNE3	GRB10	HK3	TCF19
LTF	KCNH2	PCNA	C1QA	ANKRD18A	VAT1	C1orf228
CEACAM8	CIT	CCL14	MROH6	DCN	FMO2	PADI4
HIST1H3B	HIST1H2BM	MCM4	PHGDH	FBXO5	TFRC	TIMELESS
RNASE3	HES6	FGB	FSTL3	BUB1B	FBN1	GAS6
MS4A3	APOD	GFI1	CENPA	ANKLE1	ADCY6	STOM
HIST1H1B	MYH7	CDK1	HBA1	GALNT14	NLRC4	UBE2S
CEACAM6	LPL	STMN1	TARM1	DNAH10	IGHV4-59	SLC43A1
FAM132B	CCNA2	PRG3	FAM46A	HTRA3	DZIP1L	TICRR
PRG2	UBE2C	PRC1	TCTEX1D1	FGA	FANCI	COX6A2
RETN	RP11-84C10.2	MYB	CITED4	KIF23	GSG2	MCM10
CLEC11A	SLPI	HIST1H2AG	PTTG1	MTL5	HIST1H2BE	IL1R1
BPI	CCNB2	TNC	KIF4A	RNU11	HMMR	SPARCL1
RMRP	TF	CA1	CENPF	CHL1	IARS	IGFBP4
CHIT1	PKMYT1	CLTCL1	UBE2T	NUF2	MTFR2	CENPP
RRM2	KIF2C	HIST1H4A	CENPE	HMBS	HIST1H2AE	ALDH4A1
HIST1H3J	RP11-872D17.8	IGFBP5	SMC4	HIST1H2BO	ARHGAP33	CAPN3
PRSS57	TOP2A	CRYAB	CCNF	PIWIL4	CLSPN	DHCR24
LCN2	MCM2	ACTC1	HIST1H1D	NT5DC4	FEN1	MSH5
TCN1	PKLR	SERPINB10	ASPM	SERPINB1	FAM83D	HNRNPAB
ABCA13	ERG	METTL12	NDC80	KIAA1524	CHEK1	SLC28A3
RNASE2	TK1	FGFR1	MICALL2	HIST1H4C	FAM201A	C1S
ANXA3	CLEC5A	GPR84	RECQL4	COL1A1	GABBR1	MLNR
HIST1H3C	SPP1	CEBPE	CDCA7	RP11-867G2.8	CDCA2	GADD45A
TYMS	HIST1H2AH	PTX3	CKS2	SLC1A4	DHCR7	
PRRT4	OLR1	SRGN	NCAPG2	SLC22A31	KNTC1	
HIST1H3F	AURKB	SHCBP1	TACSTD2	FGG	SERPINH1	
EPX	FBLN1	DTL	ADD2	RHAG	CTSL	
CD24	IGHV4-39	PLPPR3	MCEMP1	FN1	STEAP3	
APOE	HIST1H4J	SPTA1	HIST1H1C	KIF11	GMNN	
MKI67	CDCA3	HMGB2	CEACAM1	ITGA7	MYBL2	
HP	SPAG5	KIF20A	SAA2	H1F0	PIGQ	
HIST1H2AB	ALB	IGFBP7	CTD-2116N17.1	VEGFA	DEPDC1	
HIST1H3G	HIST1H2BB	GTSE1	CDC25A	RHCE	COL6A1	
ACTA1	CDCA5	SAA1	MMP2	IGHV4-4	RAD51	
SLC2A5	KIFC1	HIST1H2BL	DEPDC1B	LDHA	TTK	
CDT1	UHRF1	KPNA2	GPX3	PKP2	SGOL2	
ATP8B4	FOXM1	CENPM	HIST1H2BG	CENPU	MATR3	
KIAA0101	KIF18B	PLK4	NOCT	IGFBP3	CENPO	
NNMT	ESPL1	HIST1H2AM	ERLIN1	E2F8	HSPB7	
HIST1H2AL	S100A12	CDCA7L	LRP3	RNASE1	MCM6	
CDC20	SPC24	CKAP2L	PLEKHH3	ARHGAP23	MTHFD1L	
BIRC5	RP4-781K5.2	HIST1H2AD	MAD2L1	COL1A2	PYCR1	
HBD	IGFBP2	AQP1	ASNS	CDC6	POLE	
C7	IGLL1	CDC45	ORC1	ATP2C2	FAR2	
HIST1H4D	H2AFX	IGLC2	NCAPG	NEK2	CST7	
C1QC	S100A8	TPX2	MS4A4A	RGL4	DOC2B	

Supplementary Table 4. List of genes enriched in whole blood compared to bone marrow

Gene ID							
CXCL8	POU2F2	DCAF12	PLEKHF1	DGAT2	ITGAL	ARHGEF40	PCED1B
TREM1	LCK	CD6	ESPN	SH2D2A	S1PR1	MYBL1	ARRB1
PHOSPHO1	PRKCH	VWCE	MKRN1	CASS4	SPECC1	CD22	RAPGEF1
TCF7	MBNL3	FCGR3B	PF4V1	R3HDM4	ASCC2	STRADB	PTCH1
SECTM1	GZMM	IL32	MYOM2	RBM23	CXCR1	MS4A1	NHSL2
MME	BCL11B	AQP9	CSRNP1	HLA-DMB	TMIGD2	BTN3A1	ABCG1
ALS2CR12	CXCL1	FLT3LG	BTN3A2	CD300A	ATP6V0E2	AKAP7	SLC11A1
PTGS2	TBX21	KIAA1324	CCR7	RANBP10	EPHA4	DYRK2	LITAF
PRF1	FCMR	YPEL3	C15orf39	AGO2	UBE2B	GNG8	CD2
PCGF5	BBOF1	PTPRA	CD8B	EIF1AY	PTAFR	CD79A	GPBAR1
DNAJC6	CD14	ITGB7	PDLIM2	OR2W3	BHLHE40	POLL	SLC15A3
SULF2	GZMH	CCL4	IDS	NCR3	CHST15	BTG2	MICAL2
CTA-363E6.6	MPEG1	KLRB1	FBXO7	ABLIM1	SRPK2	NINJ2	SWT1
PDZK1IP1	CLIC3	PPBP	LGALS2	GIMAP7	GIMAP5	KLC3	NAAA
RP4-576H24.4	CYTH4	RUNDC3A	KLF12	CCL3L3	IFIT2	GBP5	SKI
IL2RB	XKR8	CAMK4	KRT1	ZFP36	PTPRE	CARD16	BEST1
BAG1	TNFRSF1B	PTPRCAP	AUTS2	OPTN	FCER1A	EMP3	PROK2
RAB2B	LEF1	ZNF385A	HLA-DRA	DUSP1	CACNA2D4	TRIM34	ALOX15
CXCR2	PVALB	SIGLEC10	IL10RA	DPM2	CCR3	ZFYVE28	ZHX2
HLA-DPB1	CD247	IL7R	CTC-250I14.6	LBH	FAM65B	B4GALT7	RP11-598P20.5
TMEM56	ABI3	IL12RB1	CASP1	KLF2	PPM1F	NSG1	RP11-22N19.2
CD5	BIN1	NINJ1	RP11-195O1.5	NRGN	CST3	KCNA3	CTSS
GIMAP1-GIMAP5	SNCA	AC004076.9	CDC42EP2	MEFV	TNFSF12	TFEB	GLIPR1
GNLY	HLA-DPA1	ADGRE3	FGL2	MBP	RARA-AS1	ERGIC1	PPM1A
RGS2	DPEP2	SAMD3	AC090498.1	NUAK2	KRT23	SOD2	YY1AP1
CCL4L2	PSMF1	BCL2L1	CXCR3	CD8A	MMD	THBD	PREX1
ADGRE2	CPPED1	NELL2	ADIPOR1	HCAR2	CTSW	NOTCH1	PDZD4
FGFBP2	LILRA1	GBP2	WDR60	FOXO4	DNAJB2	HCAR3	RAB8A
RARA	UBAP2	ARL4C	CMTM2	ARHGAP26	ENKUR	PRSS33	STRN3
CYTIP	CD3G	RASGRP1	FOS	RGS10	APOL3	NFATC2	CD300LB
CX3CR1	MAP3K7CL	CD3E	PTPRC	ZAP70	FAM102A	MYL9	CREB5
DOK2	BCL9L	SLC7A7	AMPD2	RARRES3	CSF1R	FAXDC2	DPP4
SHISA4	FAM46C	IGSF6	UBXN6	RASSF5	SGSH	SDPR	SHISA5
CD7	GIMAP4	TNFRSF25	LIMD2	ITK	PSMB9	PPP1R16B	CXCL16
PI3	ZDHHC18	POC1B	PIK3IP1	PRDM1	VMP1	TBCEL	PRR5
CD52	SPON2	CD300E	PRR5L	HLA-DQB1	NLRP1	GZMA	NFIX
CLEC7A	SGK1	PTGS1	EPB41	VIM-AS1	CDYL	RASA3	SELENBP1
CCL5	CRIP1	NAMPT	IGF2BP3	SH2D3C	RUNX3	ABTB1	TMCC3
GATA3	TNFRSF10C	PLD4	IRF1	CSF2RB	C9orf78	FBXW7	RP11-599B13.6
BNIP3L	SPOCK2	PTPN4	TSPAN5	TRIM58	TMCC1	PLEKHB1	DUSP6
FCHO2	TRANK1	RCAN3	LDLRAP1	FCGR2A	PACS1	ALPL	TCP11L2
PILRA	TUBB1	NLRP6	AHNAK	TESPA1	PPCDC	LRRC25	EGR1
HLA-DRB5	AMICA1	PRKAG2	LYPD2	LCOR	AKNA	TNFAIP2	LGALS9
LTB	BPGM	MARCH8	OAZ2	PTGER4	SPARC	SEPT1	PLK3
EPHB1	ARHGEF3	TUBA1A	ZFP36L1	ST6GALNAC2	TGIF2	PPP2R5B	SLFN5
CD3D	GZMB	S1PR5	KLRK1	CD27	LFNG	RNASE6	
SLC43A2	HLA-DRB1	LY86	ADGRE5	XCL2	MFAP3L	RP11-53I6.2	

Supplementary Table 5. List of indicated hematopoietic cell/lineage-specific transcripts

Erythrocytes	Megakaryocytes	T-cells	T-cells	T-cells	Neutrophil	Immature Neutrophil	Mature Neutrophil
SLC4A1	ITGA2B	PDZD4	THEMIS	TRAV21	PGLYRP1	ELANE	S100A12
TF	RAB27B	TBX21	LRRN3	TRAV23DV6	LTF	PRTN3	KRT23
AVP	GUCY1B3	CHRNA3	CCR9	TRAV26-1	ATP2C2	AZU1	FCGR3B
RUNDC3A	GP6	SIRPG	PRF1	TRAV41	VNN3	CTSG	PI3
SOX6	HGD	PITPNM2	FCRL6	DBH-AS1	CRISP3	RNASE3	STEAP4
TSPO2	PF4	GZMH	TIGIT	AC011893.3	CTSG	PGLYRP1	PROK2
HBZ	CLEC1B	GZMB	ADARB2	RP11-73O6.3	OLFM4	MMP8	CXCR1
TMCC2	CMTM5	GZMK	DTHD1	TRBV10-2	KRT23		CXCR2
SELENBP1	GP9	GNLY	KLRC2	TRBV5-4	MMP8		CD177
ALAS2	SELP	CD2	TRGV10	RP11-144L1.4	ARG1		KCNJ15
EPB42	DNM3	CD160	TRGV4	LINC00987	EPX		ALPL
GYPA	LY6G6F	ELOVL4	TRBV6-1	TRBV30	PI3		
C17orf99	LY6G6D	EPHX2	TRBV9	TRBV3-1	CRISP2		
HBA2	XXbac-BPG32J3.19	SARDH	TRBV6-5	TRBV11-2	STEAP4		
RHCE	RP11-879F14.2	KLRC1	TRBV5-6	A2M-AS1	LCN2		
HBG2		FGFBP2	TRBV4-2	LINC01550	PRG3		
TRIM10		ARL5C	TRBV20-1	RP11-291B21.2	KCNJ15		
HBA1		RORC	TRBC1	TRAV1-2	ALPL		
HBM		GZMA	TRBV27	RP11-204N11.1	FCGR3B		
HBG1		SCML4	TRAV2	RP11-158G18.1	S100A12		
UCA1		EPHA1	TRAV3	RP11-415F23.3	PROK2		
GYPB		KLRF1	TRAV4	RP11-415F23.4	CXCR1		
CTD-3154N5.2		PPP1R1C	TRAV10	RP11-104L21.3	CAMP		
AC104389.1		CD8A	TRAV12-2	TRBV12-3	RNASE3		
		PPP2R2B	TRAV13-2	TRBV10-3	CEACAM3		
		TRAT1	TRAV14DV4	TRBV13	AZU1		
		CTLA4	TRAV12-3	TRBV15	ABCA13		
		MAL	TRAV17	TRBV12-4	CXCR2		
		CD8B	TRAV19		CTD-3088G3.8		
		CXCR6	TRAV20		PRTN3		
					ELANE		
					CD177		
					LINC00671		
					ORM2		
					ORM1		
					HP		
					RP11-678G14.4		

Supplementary Table 6: Multiple myeloma patients characteristics

Patient	1	2	3
Age Range	Range (52-75)		
Diagnosis	IgA lambda	IgG Kappa	IgA Kappa
Peak relevant Ig prior to treatment	0.6 g/dl	5.6 g/dl	1.4 g/dl gamma
Plasma cells at time of transplant	13%	1%	<1%
Prior treatment	Radiation, VRD	Radiation, VRD	VRD
Plasma cells after treatment	N/A	<0.5%	<1%
Relevant Ig after transplant	0.16 g/dl	0.8 g/dl	0.038 g/dl

VRD-bortezomib, lenalidomide, dexamethasone

After transplant: evaluated 60 d post-procedure

Supplementary Table 7: EPO patient characteristics

Patient	1	2	3	4	5	6	7	8	9
Age	Range (67-91)								
Chronic kidney disease stage	4	PD	4	4	3	4	3	5	3
EPO agent	Aranesp	Procrit	Aranesp	Procrit	Aranesp	Aranesp	Aranesp	Procrit	Aranesp
Creatinine concentration (md/dL)	1.8	4.1	2.7	2.3	1.3	2.4	1.1	4.5	1.5

PD- Peritoneal Dialysis

Supplementary Table 8: G-CSF patient characteristics

Patient	1	2	3
Age	Range (24-56)		

Supplementary Table 9: AML patient characteristics

Patient	1	2	3
Age		Range (66-68)	
Bone marrow blast (%)	16	3	50
Prior Therapy	Yes*	No	No
Additional information		**	

*diffuse large B-cell lymphoma

** BM biopsy revealed lack of megakaryocyte development
