# Supplementary material for: Non-invasive characterization of human bone marrow stimulation and reconstitution by cell-free messenger RNA sequencing
Source: Nat Commun. 2020 Jan 21;11:400. doi: 10.1038/s41467-019-14253-4 (PMC6972916; doi:10.1038/s41467-019-14253-4)
Supplement: Supplementary file 2 — Reporting Summary [file 41467_2019_14253_MOESM2_ESM.pdf]

nature research

Corresponding author(s): Ibarra, Nerenberg

Last updated by author(s): Nov 26, 2019

Reporting Summary

Nature Research wishes to improve the reproducibility of the work that we publish. This form provides structure for consistency and transparency in reporting. For further information on Nature Research policies, seeAuthors & Referees and theEditorial Policy Checklist .

Statistics

For all statistical analyses, confirm that the following items are present in the figure legend, table legend, main text, or Methods section.

n/a	Confirmed	
☐	☒	The exact sample size (*n*) for each experimental group/condition, given as a discrete number and unit of measurement
☐	☒	A statement on whether measurements were taken from distinct samples or whether the same sample was measured repeatedly
☐	☒	The statistical test(s) used AND whether they are one- or two-sided<br>*Only common tests should be described solely by name; describe more complex techniques in the Methods section.*
☒	☐	A description of all covariates tested
☐	☒	A description of any assumptions or corrections, such as tests of normality and adjustment for multiple comparisons
☐	☒	A full description of the statistical parameters including central tendency (e.g. means) or other basic estimates (e.g. regression coefficient) AND variation (e.g. standard deviation) or associated estimates of uncertainty (e.g. confidence intervals)
☐	☒	For null hypothesis testing, the test statistic (e.g. *F*, *t*, *r*) with confidence intervals, effect sizes, degrees of freedom and *P* value noted<br>*Give P values as exact values whenever suitable.*
☒	☐	For Bayesian analysis, information on the choice of priors and Markov chain Monte Carlo settings
☒	☐	For hierarchical and complex designs, identification of the appropriate level for tests and full reporting of outcomes
☒	☐	Estimates of effect sizes (e.g. Cohen's *d*, Pearson's *r*), indicating how they were calculated

Our web collection on statistics for biologists contains articles on many of the points above.

Software and code

Policy information about availability of computer code

Data collection	BaseSpace (Illumina) was used for FASTQ sequencing file generation
Data analysis	STAR was used for aligning sequencing reads to the transcriptome. igBLAST was used to map Ig reads to the curated Ig transcript annotations. RSEM was used for expression level quantification. DESeq2 was used for differential expression analysis. Python and R were used for statistical analysis.

For manuscripts utilizing custom algorithms or software that are central to the research but not yet described in published literature, software must be made available to editors/reviewers. We strongly encourage code deposition in a community repository (e.g. GitHub). See the Nature Research guidelines for submitting code & software for further information.

Data

Policy information about availability of data

All manuscripts must include a data availability statement. This statement should provide the following information, where applicable:

- Accession codes, unique identifiers, or web links for publicly available datasets
- A list of figures that have associated raw data
- A description of any restrictions on data availability

RNA-seq datasets have been deposited online in Sequence Read Archive (SRA) under accession numbers PRJNA517339

October 2018

Field-specific reporting

Please select the one below that is the best fit for your research. If you are not sure, read the appropriate sections before making your selection.

Life sciences study design

All studies must disclose on these points even when the disclosure is negative.

Sample size	No sample size calculation was performed. Experiments in the manuscript describe mainly longitudinal studies in human patients, where comparisons within the same individual are performed. For comparisons between different type of samples or individuals, at least three relevant samples were assessed. Since this is a proof of concept study, we did not know the effect size before hand and therefore were not able to calculate sample size.
Data exclusions	No data is excluded from the analysis
Replication	The findings were confirmed in multiple subjects per treatment. A subset of samples from control individuals were processed in replicate
Randomization	No randomization was applied as it was not relevant for the study
Blinding	No blinding was applied as it was not relevant to the study

Reporting for specific materials, systems and methods

We require information from authors about some types of materials, experimental systems and methods used in many studies. Here, indicate whether each material, system or method listed is relevant to your study. If you are not sure if a list item applies to your research, read the appropriate section before selecting a response.

Materials & experimental systems

n/a	Involved in the study	
☒	☐	Antibodies
☒	☐	Eukaryotic cell lines
☒	☐	Palaeontology
☒	☐	Animals and other organisms
☐	☒	Human research participants
☒	☐	Clinical data

Methods

n/a	Involved in the study	
☒	☐	ChIP-seq
☒	☐	Flow cytometry
☒	☐	MRI-based neuroimaging

Human research participants

Policy information about studies involving human research participants

Population characteristics	Patient characteristics are detailed in Supplementary Tables 6-9
Recruitment	Recruitment sites and selection/exclusion criteria for the different patients are described in "Methods"
Ethics oversight	All studies were approved by their respective institutional IRBs and patients consented according to submitted study protocols. Molecular Stethoscope maintained approval for blood collection and research through Western IRB Protocol #20162748, under which normal control samples were collected. In collaboration with the Scripps Cancer Center and the Blood & Marrow Transplant Program at Scripps Green Hospital, G-CSF and EPO studies were conducted under Scripps Institutional Review Board approved protocol IRB-16-6808. Our studies involving hematopoietic bone marrow transplants, for both Multiple Myeloma and Acute Myeloid Leukemia, were approved by and conducted in accordance with Scripps IRB Protocol IRB-17-6953, in collaboration with the same groups at Scripps Green Hospital.

Note that full information on the approval of the study protocol must also be provided in the manuscript.
